# Consumers’ Evaluation of Web-Based Health Information Quality: Meta-analysis

**DOI:** 10.2196/36463

**Published:** 2022-04-28

**Authors:** Yan Zhang, Yeolib Kim

**Affiliations:** 1 School of Information The University of Texas at Austin Austin, TX United States; 2 Center for Health Communication, Moody College of Communication and Dell Medical School The University of Texas at Austin Austin, TX United States; 3 Graduate School of Technology & Innovation Management School of Business Administration Ulsan National Institute of Science and Technology Ulsan Republic of Korea

**Keywords:** online health information, information quality, credibility, trust, consumer health information behavior, meta-analysis

## Abstract

**Background:**

The internet has become a major source of health information for general consumers. Web-based health information quality varies widely across websites and applications. It is critical to understand the factors that shape consumers’ evaluation of web-based health information quality and the role that it plays in their appraisal and use of health information and information systems.

**Objective:**

This paper aimed to identify the antecedents and consequences of consumers’ evaluation of web-based health information quality as a means to consolidate the related research stream and to inform future studies on web-based health information quality.

**Methods:**

We systematically searched 10 databases, examined reference lists, and conducted manual searches. Empirical studies that investigated consumers’ evaluation of web-based health information quality, credibility, or trust and their respective relationships with antecedents or consequences were included.

**Results:**

We included 147 studies reported in 136 papers in the analysis. Among the antecedents of web-based health information quality, system navigability (ρ=0.56), aesthetics (ρ=0.49), and ease of understanding (ρ=0.49) had the strongest relationships with web-based health information quality. The strongest consequences of web-based health information quality were consumers’ intentions to use health information systems (ρ=0.58) and satisfaction with health information (ρ=0.46). Web-based health information quality relationships were moderated by numerous cultural dimensions, research designs, and publication moderators.

**Conclusions:**

Consumers largely rely on peripheral cues and less on cues that require more information processing (eg, content comprehensiveness) to determine web-based health information quality. Surprisingly, the relationships between individual differences and web-based health information quality are trivial. Web-based health information quality has stronger effects on cognitive appraisals and behavioral intentions than on behavior. Despite efforts to include various moderators, a substantial amount of variance is still unexplained, indicating a need to study additional moderators. This meta-analysis provides broad and consistent evidence for web-based health information quality relationships that have been fractured and incongruent in empirical studies.

## Introduction

The internet has become a major source of health information for general consumers. However, health information quality (IQ) varies widely across websites and web applications, and the overall quality is concerning [1,2]. Low-quality information conveys incomplete, inaccurate, or outdated knowledge, which may lead users to form erroneous health beliefs and cause negative, or even detrimental, health outcomes. Owing to the immense ramifications, web-based health IQ has attracted continued attention from researchers, health care professionals, and consumers alike.

The IQ construct has been defined in a disparate fashion. Some researchers have taken an objective view, defining IQ in relation to currently accepted medical guidelines [3]. Others recognized that the evaluation of IQ is contingent on users’ tasks, goals, and value judgments [4-6] and defined IQ, from a subjective view, as users’ perceptions of IQ [7] or “fitness for use” [8]. For the purpose of this review, we adopted the view of IQ in the study by McKinney et al [7] and defined web-based health IQ as users’ perceptions of the quality of health information on the internet. In the internet context, two other concepts share this notion: credibility and trust. Credibility is often defined as perceived IQ, whereas trust denotes users’ willingness to trust web-based information [9].

Some researchers have differentiated these 3 concepts. For instance, some view IQ as a dimension of credibility or a factor that influences credibility judgment [10], whereas others view credibility as a major dimension of IQ [11]. Some view IQ [12-14] or credibility [15] as antecedents of trust, whereas others view trustworthiness as a major dimension of credibility [16]. Despite these differences, the 3 concepts are intertwined. In the literature on consumers’ web-based health information seeking, they all, to some degree, refer to consumers’ perceived quality of web-based health information [17,18]. To achieve comprehensive coverage of the literature, we included studies that used any of the 3 terms to refer to health consumers’ perceptions of web-based health IQ.

Systematic reviews concerning IQ, trust, and credibility of web-based health information have recently been published. Sun et al [19] identified the criteria and indicators that consumers use to evaluate web-based health IQ. Sbaffi and Rowley [18] identified factors that affect consumers’ trust in and the perceived credibility of web-based health information. Kim [20] identified antecedents of trust in web-based health information. On a related note, Diviani et al [17] examined the relationship between health literacy and consumer evaluation of web-based health information. These reviews provide a comprehensive view of how consumers evaluate web-based health IQ and outline categories of antecedents of web-based health IQ, such as individual factors (eg, sociodemographic and health status), source factors (eg, reputation), content factors (eg, relevance and usefulness), and design factors (eg, layout and ease of use).

However, these reviews have several limitations. First, a plethora of antecedents of web-based health IQ was identified; however, few syntheses and comparisons were performed, resulting in a rather murky view of the most influential antecedents and how they affect web-based health IQ evaluation. Second, little effort was made to amalgamate the consequences of web-based health IQ. Third, little effort was made to explain inconsistent results across studies. For example, health literacy (and education levels and other skill-based proxies for health literacy) had a significant positive effect on perceived web-based health IQ and trust in some studies [21-24] but a negative [25-27] or insignificant [28-30] effect in others. These inconsistent results indicate that web-based health IQ relationships may be moderated by contextual factors [31].

To further enhance our knowledge of the existence, nature, and magnitude of web-based health IQ relationships and elucidate the conceptual and practical significance of the concept [32], we performed a meta-analysis to address the following research questions: (1) what antecedents and consequences are relevant to consumers’ evaluations of web-based health IQ, and (2) what moderators intervene in web-based health IQ relationships?

## Methods

### Search Strategy

A systematic search of the literature published since 2000 was performed in July 2020 on 10 databases (eg, PubMed, CINAHL, and PsycINFO), using the search query *health information* AND (*credibility* OR *quality* OR *reliability* OR *trust*) AND (*online* OR *Internet* OR *web*) within the title, abstract, and keyword fields of these databases. In addition, we tracked the references of the included papers using Google Scholar. To reduce publication bias, we also searched the ProQuest dissertation and thesis database and reviewed the proceedings of several related conferences.

### Inclusion Criteria, Exclusion Criteria, and Screening

The studies included in this review were empirical studies that reported effect sizes for web-based health IQ relationships. Studies were excluded if they met the following criteria: (1) focused on health care providers, (2) used qualitative research methods, (3) studied patients’ or consumers’ perceptions of the quality of information from noninternet sources (eg, health care providers and newspapers), (4) were not independent samples, (5) did not report effect sizes, (6) only reported significant results, and (7) were not in English.

**Figure 1:**
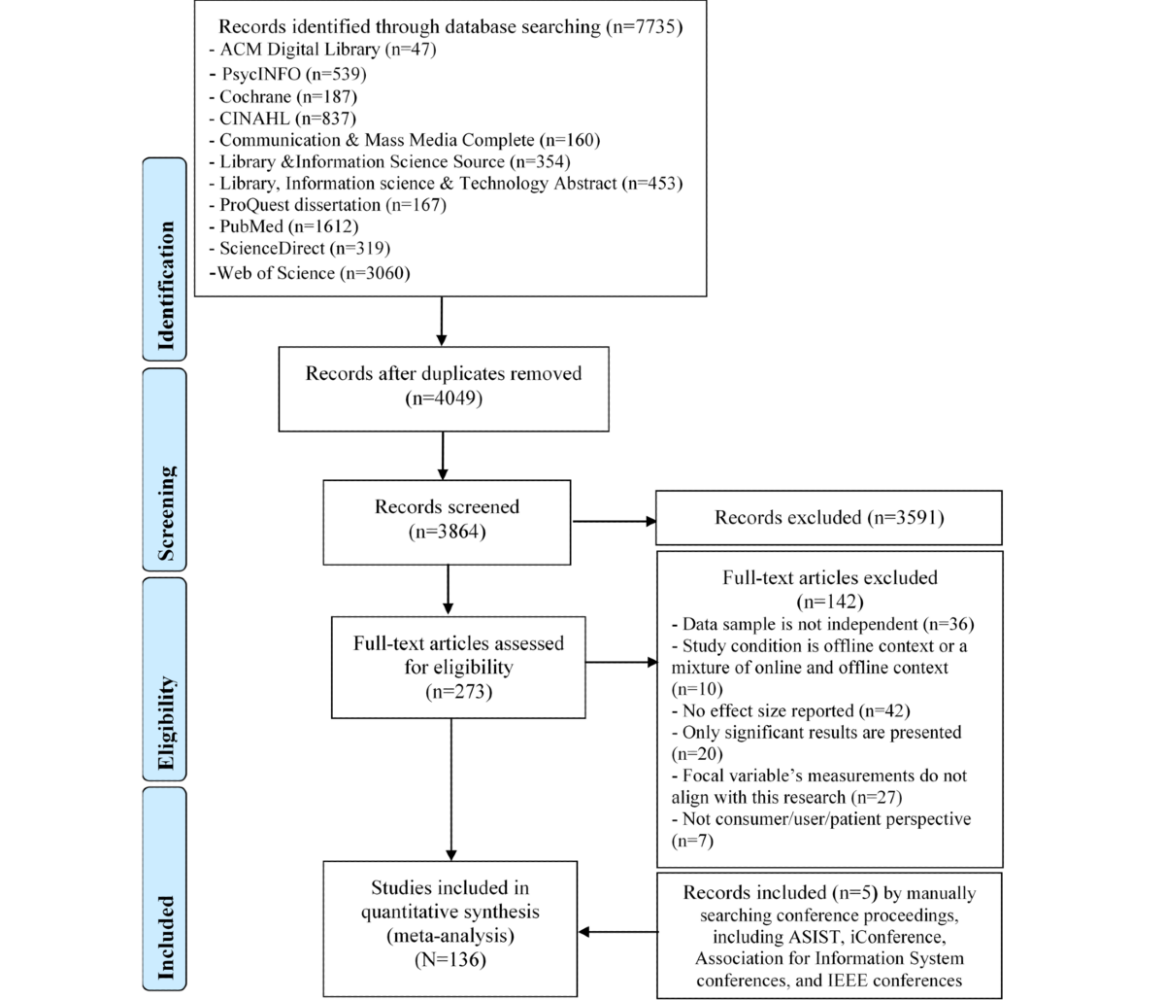
Paper screening and identification procedure.

Unique records resulting from the search were screened against the inclusion and exclusion criteria. First, two reviewers (YZ and SS) independently reviewed the titles and abstracts of the 100 randomly selected records. The full text was retrieved and perused when a decision could not be reached based on the title and abstract. The intercoder agreement was moderate (Cohen κ=0.51). Discrepancies were discussed, and we clarified the inclusion and exclusion criteria. Then, the 2 coders independently coded another 50 randomly selected records. The intercoder agreement reached 0.71. Discrepancies were discussed and resolved again. SS then screened the rest of the records. The screening was purposely kept broad to avoid missing relevant studies. The overall process resulted in 273 papers. YK reviewed the full text of these papers and further excluded 142. Relevant papers from related conference proceedings were added, resulting in a final sample of 136 papers, which reported 147 studies. The paper screening and identification procedures are illustrated in Figure 1. The list of studies is reported in Multimedia Appendix 1.

### Data Extraction and Meta-analytic Approach

One of the reviewers (YK) extracted and coded the following data from the included studies using Microsoft Excel:

1. Basic paper features: title, publication outlet, author, publication year, and publication type (journal and nonjournal)
2. Research design: stimulus type (specific vs general), technology context (social media vs nonsocial media), sample size, sample clinical status (patient vs nonpatient), sample type (student vs nonstudent), operationalization (quality vs credibility vs trust), sample year, number of instrument items, measurement reliability, sample country, sample culture dimensions, and study methods (survey vs experiment); the values for cultural dimensions were obtained by inputting the sample country into the website of Hofstede [33]
3. Antecedents and consequences: antecedent and consequence variables (when authors of the included papers used different terms to describe the same or similar concept, the terms were grouped under a preferred name; eg, the construct of direct experience with cancer in the study by Feng and Yang [34] and the construct of perceived severity of mental health in the study by McKinley and Ruppel [35] were coded as health experience and beliefs; constructs were categorized as antecedents or consequences as in the original studies), reliability scores when available, and effect sizes for specific antecedents and consequences (ie, correlations, odds ratio, β, chi-square, *F* statistic, and *t* statistic; the latter 4 were subsequently converted into correlations using formulas [36-38]).

YZ reviewed the coded data against the original full-text papers to ensure accuracy and consistency. The interrater agreement (Cohen κ) reached 0.93 for basic paper features and research design, 0.87 for grouping concepts, and 0.91 for effect sizes. Disagreements were discussed and resolved. Interested readers can contact the authors to obtain the meta-analysis database.

Following the methods by Hunter and Schmidt [36], this meta-analysis used a random-effects model to analyze correlations (*r*s). Weighted mean correlations (ρ or main effects) were computed by correcting for measurement and sampling errors. Reliabilities from each study were used to correct measurement errors. In studies that did not report a reliability value, the mean reliability (Multimedia Appendix 2) was used as the substitute. Reliability was set at 1.00 for variables assumed to have no measurement error (eg, gender, age, education, income, and race). Sample sizes were used to correct for sampling errors. Various supporting statistics such as the 95% CI, 90% credibility interval, *Q* statistic, *I*^2^ statistic, and Begg test were computed in addition to ρs. Heterogeneity was detected if the *Q* statistic was significant (*P*<.05), the *I*^2^ was >75%, or the 90% credibility interval was *wide*. The Begg test [39] exposes where publication bias exists in the meta-analysis via funnel plot asymmetry, whereby *P*<.05 implicates publication bias.

Informed by prior meta-analyses on relevant topics [40-42] and the characteristics of the included studies, we examined three categories of moderators—cultural, research design, and publication—and the operationalization of web-based health IQ (quality, credibility, and trust), resulting in a total of 13 factors (Table 1).

All moderators were categorical; thus, subgroup analyses using a random-effects model [43] were conducted to calculate the mean ρs. *Q_M_*, an omnibus test, was calculated to statistically compare subgroup means. Antecedents and consequences with a sufficient number of observations (≥20) were analyzed against moderators. Those without sufficient observations were combined into composite variables for the analysis based on conceptual similarities. The metafor package [44] in R was used to analyze the main and moderating effects.

**Table 1:** Moderators for web-based health information quality relationships.

Moderator		Definition and operationalization
**Sample culture**		This refers to the culture that sample participants belong to. It is operationalized by 5 cultural dimensions outlined in the cultural dimension theory by Hofstede [45].
	Individualism versus collectivism	Individualism is “a preference for a loosely-knit social framework” where people are supposed to take care of only themselves or their close family members [46]. Collectivism represents a preference for in-group loyalties. People in a collectivistic society must unconditionally be in service to other in-group members to show their loyalty [47].
	Power distance	This refers to the degree to which “the less powerful members of a society accept and expect that power is distributed unequally” [46]. In a society with high power distance, people “accept a hierarchical order in which everybody has a place and which needs no further justification.” In a society with low power distance, people strive to “equalize the distribution of power and demand justification for inequalities of power” [46].
	Uncertainty avoidance	This expresses “the degree to which members of a society feel uncomfortable with uncertainty and ambiguity.” Societies with strong uncertainty avoidance are “intolerant of unorthodox behavior and ideas” [46], whereas societies with weak uncertainty avoidance exhibit a more relaxed attitude [48].
	Long-term versus short-term orientation	A society with a long-term orientation fosters virtues oriented toward future rewards, in particular, perseverance and thrift [48]. A society with a short-term orientation “prefers to maintain time-honored traditions and norms while viewing societal change with suspicion” [46].
	Indulgence versus restraint	Indulgence stands for a society’s tendency to allow “relatively free gratification of basic and natural human desires related to enjoying life and having fun.” Restraint stands for “a society that suppresses gratification of needs and reregulate it by strict social norms.” [46,48].
**Research design**		This refers to the study’s research methods to address research problems, including research settings, data collection, measurement, and the analysis of data.
	Technology context	This refers to the internet technology platforms where a study situates their examination of web-based health information quality. The technology context was categorized into social media (eg, web-based health communities, Twitter, and Facebook) and non–social media (ie, general health websites).
	Sample type	This refers to whether a sample comprises students or nonstudents.
	Sample clinical status	This refers to people who assume to have no specific conditions or patients who have been diagnosed with particular conditions.
	Study methods	This refers to the research methods that a study used to collect data. Two specific research methods were frequently used and thus coded for this meta-analysis: survey and experiment.
	Stimulus type	This refers to the stimuli used in the included studies. Two types of stimuli were identified: general and specific. General stimuli are web-based health information in general (without specifications of information source and content). Specific stimuli are specific health information or health information systems (eg, a specific health website or a specific health message).
**Publication**		
	Publication outlet	This refers to the venue where a study was published. Two types of publication outlets were defined: journal and nonjournal (including conference proceedings and theses and dissertations).
	Time	This refers to when a study was published. Two periods were defined—before 2014 and in or after 2014—by applying the median split on the publication year.
Operationalization of web-based health information quality		This refers to the three focal concepts included in the analysis: web-based health information quality, credibility, and trust in web-based health information.

## Results

### Basic Characteristics of the Included Papers

The 136 papers included 109 (80.1%) journal articles, 20 (14.7%) conference papers, and 7 (5.2%) theses and dissertations. The publication years ranged from 2000 to 2020, with 75% (102/136) of the papers published after 2010. The health domains covered included both general and specific health topics (eg, schizophrenia, cancer, HIV, and prescription medications).

The included papers reported 147 independent studies. Sample sizes ranged from 34 to 8586 (median 252); 67.3% (99/147) of samples involved nonstudents, 32.7% (48/147) involved students, 8.8% (13/147) of samples were patients, and 91.2% (134/147) were nonpatients. Among the 133 samples that reported countries (15 countries), 76 (57.1%) were from the United States, followed by 16 (12%) from China, 10 (7.5%) from Korea, 8 (6%) from Germany, and 5 (3.8%) from Australia.

### Antecedents and Consequences of Web-Based Health IQ

Table 2 presents 18 antecedents and 8 consequences of web-based health IQ with at least 6 observations. Those with the number of samples <6 were not included in the analysis as the results tend to be less generalizable [41]. The antecedents fell into four categories: individual difference, source, content, and design. The consequences fell into three categories: cognitive appraisals, behavioral intentions, and behaviors.

Table 3 presents the main effects of the antecedents and consequences. Using the Cohen criteria [49] for judging the magnitude of correlation effect sizes, the design factor—navigability—was most strongly related to web-based health IQ (ρ=0.56), followed by the other design factor—aesthetics (ρ=0.49)—and a content factor—ease of understanding (ρ=0.49). Four other factors—source trustworthiness (ρ=0.28), health knowledge (ρ=0.15), internet experience (ρ=0.13), and social endorsement (ρ=0.10)—showed significant but weak relationships with web-based health IQ.

On the basis of the Begg test, which takes into account publication bias (Begg *P*=.02), and using the trim-and-fill method [50] with 10 imputed studies on the right side of the funnel plot, age had a significant association with web-based health IQ (ρ=0.27; 95% CI 0.06-0.48; Q=3753.86). Thus, the age and web-based health IQ relationship changed from nonsignificant to significant, with individuals who were older rating the web-based health IQ higher than those who were younger. The remaining factors were not significantly related to web-based health IQ.

Regarding consequences, the web-based health IQ exerted the strongest effect on intentions to use health information systems (ρ=0.58). Its relationship with intentions to use health information was also significant but not as strong (ρ=0.37). Web-based health IQ’s relationships with cognitive appraisal factors were mostly moderate, with the effect size for satisfaction being the largest (ρ=0.46). Web-based health IQ was moderately related to health information seeking (ρ=0.30) and did not have a significant relationship with health information use.

Across the results of the main effects, Q statistics were *substantial*, indicating that the effect size distribution was heterogeneous and that some variables other than subject-level sampling and measurement errors contributed to the effect size variances [51]. Confirming the Q statistics, the *I*^2^ statistics indicated wide dispersion. The credibility interval for all relationships was wide, further implying that the effect size distribution was heterogeneous.

**Table 2:** Antecedents and consequences of web-based health information quality with at least 6 observations.

Variable				Definition
**Antecedents**				
	**Individual differences**			
		Gender	The gender of the participants included in study samples (female=1 and male=0)	
		Age	The age of the participants included in study samples	
		Education	The education levels of the participants included in study samples	
		Income	The income of the participants included in study samples	
		Race	The race of the participants included in study samples (White=1 and non-White=0)	
		Internet experience	An individual’s experience with using the internet, as manifested in aspects such as the length or frequency of use and the use of the range of web-based services [52]	
		Personal involvement	An individual’s perceived personal relevance of the web-based health information [53]	
		Perceived health status	Individuals’ self-assessment of the status of their overall personal physical and mental health [54,55]	
		Condition experience and beliefs	An individual’s experience with a health condition, perceived risk for developing the condition, and perceived severity of the condition	
		Health literacy	Individuals’ ability to obtain health information from both electronic and nonelectronic sources and their ability to process, understand, and apply the obtained health information to solve health problems and make appropriate health decisions [56,57]	
		Health knowledge	Individuals’ knowledge about their health problems and the care for the problems [58]	
	**Source-related factors**			
		Source trustworthiness	The extent to which an individual believes that a specific web-based health information provider has attributes (eg, reputation) that are beneficial to the consumer [14]	
		Source expertise	The extent to which the source or the author of a message, webpage, or website is perceived to be capable of making correct assertions [59]	
	**Content factors**			
		Ease of understanding	Whether the provided information is easy to understand (eg, in everyday language) and informative to users [60,61]	
		Social endorsement	Endorsements from other users of a website and could be manifested in forms ranging from sharing, commenting, and rating to liking [62]	
		Content comprehensiveness	Whether information provided is comprehensive, providing users with comparatively complete information (eg, necessary information to establish a medical claim, statistics, references, testimonials, source and author information, and user support information) [63,64]	
	**Design factors**			
		Navigability	Whether a website has clear navigation menus and effective hyperlinks and whether the information is easy to access by searching or browsing [65,66]	
		Aesthetics	The visual design of a website, including the structural features such as typography, images, color, and aesthetics (eg, whether the website is professional and appealing) [67]	
**Consequences**				
	**Cognitive appraisals**			
		Attitudes	Individuals’ evaluations of and feelings about health websites or web-based health information [64,68]	
		Perceived usefulness	The degree to which consumers believe that using health information on the internet would enhance their health-related activities [47]	
		Perceived health benefits	The perceived level of rewards or risks that people have about the consequences of using or acting on web-based health information [14,65]	
		Satisfaction with health information	Individuals’ satisfaction with health websites or web-based health information	
	**Behavioral intentions**			
		Intentions to use health information	Individuals’ intentions or willingness to use web-based health information to make health decisions, manage health problems, or inform health behaviors	
		Intentions to use health information systems	Individuals’ intentions or willingness to use web-based health information systems to seek health information	
	**Behavior**			
		Health information seeking	Individuals’ use of web-based or offline sources to find health-related information, which is manifested in aspects such as the types of information sought and the frequency and intensity of health information seeking	
		Use health information	Individuals’ use or application of health information (from web-based or offline sources) to make health decisions, manage health problems, or inform health behaviors	

**Table 3:** Antecedents and consequences of quality of web-based health information.

Factors			Samples, n	Sample size, N	r^a^	ρ^b^, mean (SD)	95% CI	90% CV^c^	Q^d^	*I*^2^ (%)^e^	Begg *P* value^f^
**Antecedents**											
	**Individual differences**										
		Gender (female)	25	20,101	0.04	0.04 (0.13)	–0.04 to 0.13	–0.17 to 0.25	216.29^g^	86.45	.22
		Age	20	23,463	0.04	0.04 (0.13)	–0.11 to 0.20	–0.18 to 0.26	834.84^g^	97.15	.02
		Education	15	16,874	0.05	0.05 (0.17)	–0.09 to 0.20	–0.23 to 0.33	332.32^g^	94.29	.70
		Internet experience	14	6235	0.12	0.13 (0.15)	0.01 to 0.24	–0.11 to 0.37	186.18^g^	91.99	.75
		Personal involvement	13	4171	0.11	0.13 (0.22)	–0.05 to 0.31	–0.23 to 0.49	465.37^g^	97.08	.31
		Perceived health status	9	26,207	0.04	0.04 (0.21)	–0.12 to 0.19	–0.31 to 0.39	715.44^g^	98.55	.92
		Income	9	18,177	0.05	0.05 (0.10)	–0.08 to 0.19	–0.11 to 0.21	270.04^g^	95.65	.61
		Race (White)	9	14,162	–0.07	–0.08 (0.26)	–0.52 to 0.37	–0.51 to 0.35	2609.14^g^	99.56	.08
		Condition experience and beliefs	7	7772	0.05	0.05 (0.10)	–0.01 to 0.11	–0.12 to 0.22	18.74^g^	48.88	.99
		Health literacy	6	3661	0.18	0.22 (0.28)	–0.01 to 0.45	–0.25 to 0.69	298.22^g^	97.83	.27
		Health knowledge	6	2797	0.13	0.15 (0.11)	0.07 to 0.22	–0.03 to 0.33	18.26^g^	63.64	.72
	**Source-related factors**										
		Source trustworthiness	17	4154	0.25	0.28 (0.25)	0.10 to 0.45	–0.14 to 0.70	950.38^g^	97.78	.66
		Source expertise	13	5988	0.17	0.20 (0.27)	–0.08 to 0.49	–0.25 to 0.65	649.35^g^	97.66	.44
	**Content-related factors**										
		Ease of understanding	14	3981	0.41	0.49 (0.28)	0.35 to 0.63	0.03 to 0.95	698.28^g^	97.90	.47
		Social endorsement	7	2267	0.09	0.10 (0.17)	0.00 to 0.19	–0.18 to 0.38	32.03^g^	77.57	.14
		Content comprehensiveness	7	1373	0.18	0.21 (0.30)	–0.11 to 0.54	–0.29 to 0.71	549.04^g^	97.57	.56
	**Design-related factors**										
		Navigability	12	3099	0.47	0.56 (0.33)	0.44 to 0.67	0.02 to 1.00	436.08^g^	96.85	.21
		Aesthetics	11	4307	0.40	0.49 (0.26)	0.30 to 0.68	0.06 to 0.92	434.41^g^	95.99	.06
**Consequences**											
	**Cognitive appraisals**										
		Attitudes	14	3934	0.38	0.43 (0.24)	0.36 to 0.50	0.04 to 0.82	1871.08^g^	88.04	.75
		Perceived usefulness	10	11,110	0.25	0.29 (0.23)	0.08 to 0.50	–0.09 to 0.67	557.55^g^	97.78	.60
		Perceived health benefits	9	6292	0.32	0.37 (0.16)	0.26 to 0.49	0.10 to 0.64	189.17^g^	94.86	.61
		Satisfaction with health information	9	3334	0.41	0.46 (0.29)	0.41 to 0.51	–0.01 to 0.93	1699.85^g^	84.31	.36
	**Behavioral intentions**										
		Intentions to use health information	17	7663	0.32	0.37 (0.33)	0.32 to 0.43	–0.18 to 0.92	4162.85^g^	99.23	.66
		Intentions to use health information systems	8	1614	0.49	0.58 (0.24)	0.43 to 0.72	0.19 to 0.97	290.34^g^	95.73	.55
	**Behavior**										
		Health information seeking	18	26,259	0.25	0.30 (0.28)	0.15 to 0.46	–0.16 to 0.76	12,308.96^g^	99.59	.08
		Health information use	15	15,021	0.21	0.25 (0.28)	–0.00 to 0.50	–0.21 to 0.71	1083.33^g^	98.36	.77

^a^Weighted mean correlation.

^b^Corrected weighted mean correlation and SD of ρ.

^c^90% credibility interval.

^d^Heterogeneity statistic.

^e^Percentage of variation across studies that is because of heterogeneity.

^f^The Begg test for funnel plot asymmetry.

^g^*P*<.01.

### Moderators of Web-Based Health IQ Relationships

Substantial heterogeneity calls for moderator analyses to explain the variance. The examined moderators included culture, research design, publication factors, and one operationalization-related moderator—the focal variable. The analysis was performed on web-based health IQ’s relationships with eight factors: two individual factors—gender and age—enabled by adequate sample numbers and six composite factors—source, content, design, cognitive appraisals, behavioral intentions, and behavior—formed by combining lower-level antecedents and consequences to offer adequate observations for the analysis. For the moderator analysis involving age and web-based health IQ, we did not include the 10 imputed studies, given that incorporating simulated data can distort the subgroup comparison. Table 4 presents the subgroup mean values and Q_M_ statistics. Other relevant statistics (95% CI, 90% credibility interval, Q_E_, and *R*^2^*)* can be found in Multimedia Appendices 3-10. All moderators were significantly related to the effect size of at least one web-based health IQ relationship examined; 6 moderators significantly affected ≥3 relationships. The following interpretations focused on subgroups with significant differences.

Culture moderated the three antecedents of web-based health IQ: gender, age, and source. Females in individualistic (ρ=0.06 vs ρ=–0.11), low power distance (ρ=0.06 vs ρ=–0.02), and high uncertainty avoidance (ρ=0.08 vs ρ=–0.03) cultures rated web-based health IQ higher than males. Older individuals in low uncertainty avoidance (ρ=0.21 vs ρ=–0.07) and indulgence cultures (ρ=0.19 vs ρ=–0.05) rated web-based health IQ higher. Individuals with high uncertainty avoidance (ρ=0.37 vs ρ=0.20), long-term orientation (ρ=0.32 vs ρ=0.19), and restraint (ρ=0.37 vs ρ=0.17) cultures exhibited a stronger source and web-based health IQ relationship.

Culture moderated two consequences of web-based health IQ: cognitive appraisals and behavioral intentions. Individuals in long-term cultures had higher cognitive appraisals of web-based health IQ (ρ=0.40 vs ρ=0.27). Individuals with low uncertainty avoidance (ρ=0.59 vs ρ=0.41), short-term orientation (ρ=0.80 vs ρ=0.43), and indulgence cultures (ρ=0.60 vs ρ=0.43) had higher behavioral intentions as a result of the web-based health IQ than individuals in their respective counterpart cultures.

Research design moderated two antecedents of web-based health IQ: gender and content. Women rated the web-based health IQ higher in studies using the survey method (ρ=0.06 vs ρ=–0.06), non–social media technology context (ρ=0.06 vs ρ=–0.10), and nonpatient samples (ρ=0.09 vs 0.00). The content and web-based health IQ relationships were stronger in studies using the survey method (ρ=0.51 vs ρ=0.21), general stimuli (ρ=0.73 vs ρ=0.30), and nonstudent samples (ρ=0.43 vs ρ=0.24).

Research design moderated three consequences of web-based health IQ: cognitive appraisals, behavioral intentions, and behavior. Studies using specific stimuli (ρ=0.48 vs ρ=0.27) and nonstudent samples (ρ=0.35 vs ρ=0.26) produced larger effect sizes for the web-based health IQ and cognitive appraisals relationship. Studies using specific stimuli (ρ=0.54 vs ρ=0.32), social media context (ρ=0.45 vs ρ=0.39), and student samples (ρ=0.53 vs ρ=0.39) reported higher behavioral intentions. Student samples also produced a larger effect size for the web-based health IQ and behavior relationship (ρ=0.66 vs ρ=0.20).

Publication factors moderated the gender and web-based health IQ relationship. Journal articles (ρ=0.06 vs ρ=–0.08) and papers published before 2014 (*ρ*=0.08 vs ρ=–0.01) reported larger effect sizes than their respective counterparts. Publication year moderated web-based health IQ and cognitive appraisals and web-based health IQ and behavioral intentions, with recent publications (2014 and after) reporting lower cognitive appraisals (ρ=0.32 vs ρ=0.37) but higher behavioral intentions (ρ=0.55 vs ρ=0.25).

The three focal variables—quality, credibility, and trust—produced significant differences in 2 web-based health IQ relationships. The quality subgroup reported a stronger design and web-based health IQ relationship than the credibility subgroup (ρ=0.58 vs ρ=0.33). The omnibus test for comparing the focal variables in the web-based health IQ and behavioral intentions was significant (Q_M_=30.50; *P*<.01). Post hoc tests revealed that the significant difference was because of the trust group being higher than the quality group (Q_M_=13.63; *P*<.01) and the trust group being higher than the credibility group (Q_M_=26.85; *P*<.01).

**Table 4:** Influence of moderators on quality of web-based health information relationships.

Moderators				Gender (female=1)		Age		Source-related factors		Content-related factors		Design-related factors		Cognitive appraisals		Behavioral intentions		Behavior
**Culture**																		
	**Individualism versus collectivism, Q_M_^a^**			10.50^b^		0.32		3.20		0.03		0.24		1.88		2.71		1.67
		Individualism, mean (k; N)^c^	0.06 (18; 17,745)		0.05 (12; 19,834)		0.22 (21; 7645)		0.37 (20; 5491)		0.39 (9; 2127)		0.31 (23; 15,533)		0.43 (13; 4684)		0.27 (20; 33,688)	
		Collectivism, mean (k; N)	–0.11 (4; 1242)		0.03 (5; 2542)		0.44 (6; 1460)		0.46 (4; 468)		0.57 (6; 1917)		0.39 (11; 4008)		0.58 (7; 1977)		0.37 (9; 5515)	
	**Power distance, Q_M_**			3.85^d^		0.07		3.20		0.03		0.24		1.88		2.71		1.67
		High, mean (k; N)	–0.02 (5; 1901)		0.01 (6; 3201)		0.44 (6; 1460)		0.46 (4; 468)		0.57 (6; 1917)		0.39 (11; 4008)		0.58 (7; 1977)		0.37 (9; 5515)	
		Low, mean (k; N)	0.06 (17; 17,086)		0.05 (11; 19,175)		0.22 (21; 7645)		0.37 (20; 5491)		0.39 (9; 2127)		0.31 (23; 15,533)		0.43 (13; 4684)		0.27 (20; 33,688)	
	**Uncertainty avoidance, Q_M_**			6.78^b^		7.37^b^		6.25^d^		0.13		0.25		1.23		109.01^b^		2.02
		High, mean (k; N)	0.08 (10; 13,180)		–0.07 (8; 12,795)		0.37 (14; 3022)		0.45 (11; 2177)		0.61 (4; 969)		0.31 (17; 7284)		0.41 (9; 4344)		0.42 (12; 14,139)	
		Low, mean (k; N)	–0.03 (12; 5807)		0.21 (9; 9581)		0.20 (13; 6083)		0.33 (13; 3782)		0.43 (11; 3075)		0.33 (17; 12,257)		0.59 (11; 2317)		0.21 (17; 25,064)	
	**Orientation, Q_M_**			0.57		2.93		7.21^b^		0.52		0.02		3.88^d^		457.96^b^		.08
		Long-term, mean (k; N)	0.07 (15; 14,013)		–0.05 (13; 14,411)		0.32 (19; 4457)		0.48 (15; 3551)		0.52 (9; 2556)		0.40 (23; 7935)		0.43 (15; 5761)		0.29 (17; 13,738)	
		Short-term, mean (k; N)	–0.01 (7; 4974)		0.22 (4; 7965)		0.19 (8; 4648)		0.22 (9; 2408)		0.39 (6; 1488)		0.27 (11; 11,606)		0.80 (5; 900)		0.28 (12; 25,465)	
	**Indulgence versus restraint, Q_M_**			0.00		3.86^d^		11.47^b^		0.13		0.04		0.37		165.28^b^		0.92
		Indulgence, mean (k; N)	0.00 (10; 6213)		0.19 (6; 8974)		0.17 (9; 5223)		0.34 (11; 3582)		0.41 (7; 1862)		0.31 (13; 11,190)		0.60 (8; 1704)		0.20 (14; 22,776)	
		Restraint, mean (k; N)	0.07 (12; 12,774)		–0.05 (11; 13,402)		0.37 (18; 3882)		0.43 (12; 2307)		0.53 (8; 2182)		0.38 (20; 6945)		0.43 (12; 4957)		0.29 (12; 11,502)	
**Methods**																		
	**Study method, Q_M_**			5.39^d^		0.01		0.57		9.55^b^		2.46		0.49		3.39		N/A^e^
		Survey, mean (k; N)	0.06 (17; 17,929)		0.04 (13; 21,681)		0.25 (7; 4884)		0.51 (10; 3271)		0.56 (14; 5795)		0.34 (27; 21,314)		0.40 (22; 8870)		—^f^	
		Experiment, mean (k; N)	–0.06 (8; 2172)		–0.01 (7; 1782)		0.22 (23; 5258)		0.21 (17; 3772)		0.26 (6; 897)		0.41 (10; 1702)		0.46 (3; 407)		N/A	
	**Stimulus type, Q_M_**			2.13		0.05		1.31		9.53^b^		0.49		4.74^a^		100.36^b^		1.04
		General, mean (k; N)	0.06 (13; 16,088)		0.05 (11; 20,648)		0.35 (4; 1321)		0.73 (5; 822)		0.60 (9; 3963)		0.27 (18; 15,477)		0.32 (11; 5688)		0.29 (25; 35,528)	
		Specific, mean (k; N)	–0.01 (12; 4013)		–0.02 (9; 2815)		0.22 (26; 8821)		0.30 (22; 6221)		0.39 (11; 2729)		0.48 (19; 7539)		0.54 (14; 3589)		0.27 (6; 3983)	
	**Technology context, Q_M_**			4.55^d^		0.06		3.33		0.72		N/A		N/A		5.18^d^		N/A
		Social media, mean (k; N)	–0.10 (4; 2092)		0.02 (3; 1397)		0.32 (10; 2011)		0.24 (10; 2493)		—		—		0.45 (6; 1107)		—	
		Non–social media, mean (k; N)	0.06 (21; 18,009)		0.04 (17; 22,066)		0.21 (20; 8131)		0.41 (17; 4550)		N/A		N/A		0.39 (19; 8170)		N/A	
	**Sample clinical status, Q_M_**			7.18^b^		N/A		N/A		N/A		N/A		N/A		0.57		0.04
		Nonpatients, mean (k; N)	0.09 (4; 9478)		—		—		—		—		—		0.24 (3; 1646)		0.11 (6; 4192)	
		Patients, mean (k; N)	0.00 (21; 10,623)		N/A		N/A		N/A		N/A		N/A		0.44 (22; 7631)		0.31 (25; 35,319)	
	**Sample type, Q_M_**			0.00		0.03		0.00		5.63^d^		.04		9.20^b^		4.69^d^		3.71^d^
		Students, mean (k; N)	0.01 (7; 1978)		–0.03 (3; 594)		0.22 (13; 2959)		0.24 (13; 2947)		0.50 (4; 841)		0.26 (8; 1693)		0.53 (6; 1133)		0.66 (9; 7008)	
		Nonstudents, mean (k; N)	0.05 (18; 18,123)		0.04 (17; 22,869)		0.24 (17; 7183)		0.43 (14; 4096)		0.52 (16; 5851)		0.35 (29; 21,323)		0.39 (19; 8144)		0.20 (22; 32,503)	
**Publication**																		
	**Outlet, Q_M_**			6.41^d^		0.02		0.18		0.73		2.85		0.41		N/A		0.01
		Journal, mean (k; N)	0.06 (17; 18,204)		0.05 (12; 21,288)		0.24 (20; 7528)		0.38 (19; 5426)		0.50 (13; 4751)		0.33 (30; 21,454)		—		0.29 (26; 38,726)	
		Nonjournal, mean (k; N)	–0.08 (8; 1897)		–0.01 (8; 2175)		0.21 (10; 2614)		0.26 (8; 1617)		0.54 (7; 1941)		0.45 (7; 1562)		N/A		0.22 (5; 785)	
	**Year, Q_M_**			5.26^d^		0.15		0.00		0.01		0.21		6.42^d^		146.34^b^		0.00
		Before 2014, mean (k; N)	0.08 (9; 12,145)		0.07 (7; 16,892)		0.26 (13; 5463)		0.33 (8; 1684)		0.52 (12; 4380)		0.37 (21; 14,357)		0.25 (10; 4430)		0.27 (15; 24,416)	
		2014 and after, mean (k; N)	–0.01 (16; 7956)		–0.02 (13; 6571)		0.20 (17; 4679)		0.36 (19; 5359)		0.52 (8; 2312)		0.32 (16; 8659)		0.55 (15; 4847)		0.31 (16; 15,095)	
**Focal variable, Q_M_**				0.07		0*.*94		0.09		2.19		9.34^b^		3.59		30.50^b^		4.12
		Quality, mean (k; N)	—		0.04 (3; 737)		0.30 (8; 1899)		0.31 (6; 1632)		0.58 (10; 4815)		0.45 (11; 4510)		0.40 (7; 3355)		0.40 (6; 4095)	
		Credibility, mean (k; N)	0.02 (9; 2867)		0.22 (8; 7801)		0.20 (18; 4859)		0.29 (16; 3943)		0.33 (9; 1704)		0.26 (17; 9301)		0.31 (8; 1577)		0.19 (7; 7341)	
		Trust, mean (k; N)	0.06 (9; 14,572)		–0.05 (8; 14,290)		0.25 (4; 3384)		0.57 (5; 1468)		—		0.36 (9; 9205)		0.45 (10; 4345)		0.30 (17; 27,889)	

^a^Omnibus test comparing group means.

^b^*P*<.01.

^c^Cell entries show subgroup means (weighted mean correlation corrected for measurement unreliability); each parenthesis contains *k* (number of samples) and N (total sample size).

^d^*P*<.05.

^e^N/A: not applicable; insufficient effect sizes for subgroup comparison.

^f^Not available.

## Discussion

Using a comprehensive meta-analytic approach, this study analyzed antecedents and consequences of consumer web-based health IQ evaluations and contextual factors that moderate the relationships based on 147 independent studies. The major findings are discussed in the following sections.

### Web-Based Health IQ Antecedents

Consistent with systematic reviews of consumer web-based health information evaluation behavior [18,19], we identified four major categories of antecedents of web-based health IQ: individual, source, content, and design factors. Furthermore, we revealed the magnitude of the antecedents’ effect. We found that among the 18 antecedents examined, navigability (design) was the strongest predictor of web-based health IQ, followed by ease of understanding (content) and aesthetics (design). Four factors had significant but weak relationships with web-based health IQ: source trustworthiness (source), health knowledge (individual), internet experience (individual), and social endorsement (content). Age (individual) was significantly related to web-based health IQ after correcting for publication bias. However, this result needs to be viewed with caution as imputed data were generated to obtain this result. The remaining 10 antecedents were not substantially related to web-based health IQ evaluation.

These results suggest that consumers rely prominently on peripheral cues (eg, navigability, aesthetics, and ease of understanding) and less on systematic cues (eg, content comprehensiveness) to evaluate web-based health IQ. This is consistent with the Fogg et al [69,70] findings from large-scale surveys that website design look and ease of use (including navigability) were the most prominent influencers of website credibility, exerting stronger impacts than source expertise and trustworthiness. According to dual processing models of information processing and assessment, such as the Elaboration Likelihood Model and Heuristic-Systematic Model [71,72], these results can be attributed to consumers’ lack of motivation and/or ability to evaluate web-based health IQ [73,74]. However, the results were not conclusive. First, it is possible that theoretically significant motivational and ability factors, such as personal involvement and source expertise [71,72], did not show a significant direct impact on web-based health IQ in this research because their relationships were moderated by contextual factors, which were not analyzed because of insufficient observations. Second, other theoretically and/or empirically significant influencers of web-based health IQ that are closely related to systematic information processing, such as augment strength [14] and content consistency [75,76], were not analyzed because of insufficient observations; thus, their effects were not accounted for in this research. More research is needed to elucidate the antecedent and web-based health IQ relationships.

### Consequences

Web-based health IQ was significantly related to all the consequences identified in the research, except for health information use. The effect of web-based health IQ on behavioral intentions (particularly intentions to use health information systems) was the strongest, followed by cognitive appraisal factors (particularly satisfaction with health information). The relationship of web-based health IQ with health information–seeking behaviors was moderate, consistent with the findings of another meta-analysis of credibility and health information seeking [77].

The information system success model posits that IQ predicts users’ intention to use or use of and satisfaction with an information system [78,79]. The model of information adoption posits that IQ determines users’ attitudes toward information (ie, usefulness) [80,81]. Empirical research in information systems has provided strong support for the IQ-satisfaction relationship [40], whereas support for the IQ-use relationship has been mixed [79]. Our meta-analyses of web-based health IQ consequences are largely consistent with these findings, suggesting that web-based health IQ is important for consumers’ intentions to use and satisfaction with web-based health information systems and information and information-seeking behavior. The 2 aforementioned models, although primarily developed and tested in organizational or individual work settings, are applicable in the context of consumers’ web-based health information seeking.

### Moderators

Culture moderated three antecedent and web-based health IQ relationships (ie, age, gender, and source) and two web-based health IQ and consequence relationships (ie, cognitive appraisals and behavioral intentions), demonstrating itself as an important factor shaping both web-based health IQ evaluation and its consequences. However, few empirical studies have directly examined the culture and web-based health IQ relationships. Future studies should fill this gap, which is critical for informing the design of health information systems and policies that serve different cultural groups in and across nations.

Research design factors moderated two antecedent and web-based health IQ (ie, gender and content) and all 3 consequence and web-based health IQ relationships, reinforcing the importance of careful research design in studying web-based health IQ. It is worth noting that sample type and stimulus type affected the greatest number of relationships, with student samples and studies using general stimuli reporting stronger content and web-based health IQ relationships and having lower cognitive appraisals but stronger behavioral intentions (and stronger behavior for the student samples). The results caution the use of student samples and general stimuli when studying web-based health IQ relationships. The clinical status of the sample moderated the gender and web-based health IQ relationship. It may moderate more relationships for patients’ personal involvement [72]; however, it remains inconclusive because of insufficient observations.

Limited publication venue bias was observed as the publication outlet moderated only the gender and web-based health IQ relationship. As a proxy to detect how web-based health IQ relationships have fluctuated over time, the publication year moderated three relationships—gender and web-based health IQ, web-based health IQ and cognitive appraisals, and web-based health IQ and behavioral intentions—revealing that individuals’ cognitive appraisals of web-based health IQ lessened; however, intentions to act on the information increased over time. It is plausible that consumers are becoming more critical as arbiters of web-based health information; however, they are also becoming more receptive to web-based health information and information systems.

The focal variables (credibility, trust, and quality) moderated two relationships—design and web-based health IQ and web-based health IQ and behavioral intentions—out of the 8 relationships examined, indicating that some theoretical and/or methodological issues exist that promulgate this effect size disparity. Studies using quality identified a larger effect size than studies that used credibility in the design and web-based health IQ relationship. This can be attributed to the fact that studies that examined the relationship viewed quality as intrinsic merit of information (eg, accuracy, argument strength, consistency, and comprehensiveness) [14,82] and credibility as perceived reliability or trustworthiness of information [64]. In such a case, we speculate that consumers had more difficulty determining IQ than credibility [83]; thus, they need to rely more on design factors to form IQ perceptions. For the web-based health IQ and behavioral intentions relationship, studies using trust produced the largest effect size, followed by studies using quality and credibility, indicating that trust most strongly predicts behavioral intentions, followed by quality and credibility. This may be because studies on web-based health IQ and behavioral intentions were more likely to consider risk and gain assessment as part of the trust formation process [84-86], such that trust showed a higher predictive power for behavioral intentions [12,14].

### Limitations

As with all meta-analysis studies, the main effects of a small number of observations or small sample size (eg, race or health knowledge with web-based health IQ relationships) should be interpreted with caution. Insufficient observations also limit moderator analyses, whereby moderator analyses of some theoretically or practically important relationships (eg, race, personal involvement, and health literacy with web-based health IQ relationships) were not performed. Relatedly, some antecedents and consequences were combined to form high-level categories to enable moderator analyses, which inevitably masks how some important specific relationships (eg, web-based health IQ and use of health information) might be affected by moderators.

In terms of moderator analysis, consistent with prior meta-analysis findings, student-based results were biased [79], and survey-based results produced larger effect sizes than experience-based results [41]. The most noteworthy finding concerning moderator analyses was that the three conceptualizations of web-based health IQ (ie, quality, credibility, and trust) moderated two out of the eight relationships examined (ie, design and web-based health IQ and web-based health IQ and behavior intentions), suggesting that despite a significant conceptual overlap, theoretical and/or operationalization differences exist among the 3 constructs. This result should be interpreted in light of the fact that we took a phenomenological approach, adopting the authors’ conceptualizations of web-based health IQ (quality, credibility, and trust). A detailed examination of the definitions and measures of these constructs is warranted to elucidate the differences among the concepts. A preliminary examination of the included papers revealed that not many studies provided explicit definitions of the constructs and that measures of the same construct varied, with many articles not including specific and complete measures. These observations call for future empirical studies to offer clearer definitions of the constructs and complete measures to enable a fair assessment of these concepts for future literature synthesis.

Despite attempts to apply various moderators to explain the variance across web-based health IQ relationships, substantial variance remained. Future research should prudently select additional moderators to explain this variance. For example, health topics merit investigation as an important contextual factor with theoretical significance for studying information-seeking behavior [87,88]. Website type also merits investigation in light of recent findings that it influences how consumers apply content, design, and source factors to evaluate web-based health IQ [89,90].

### Conclusions

On the basis of a meta-analysis of 147 empirical studies, our study confirmed that consumers’ evaluation of web-based health IQ significantly affects their cognitive appraisals of web-based health information, intentions to use web-based information systems and information, and information-seeking behavior, suggesting the important role that web-based health IQ plays in promoting health information seeking. The study also confirmed that consumers’ evaluation of web-based health IQ is shaped by source, content, design, and individual factors, with the most influential factors being design, particularly navigability and aesthetics, and ease of understanding of content. Many individual factors, such as gender, race, education, personal involvement, and health literacy, did not show significant relationships with web-based health IQ. However, moderator analyses and the residual variance after the analyses suggest that these relationships may be moderated by numerous methodological and nonmethodological moderators. Patient empowerment and active participation in health care require individuals to have equal access to high-quality health information. More studies are needed to elucidate individual factors and web-based health IQ relationships to address potential information access disparities among different user groups.

## Appendix

Multimedia Appendix 1 List of studies included in sample.

Multimedia Appendix 2 Mean reliabilities.

Multimedia Appendix 3 Influence of moderators on the relationship between gender and web-based health information quality.

Multimedia Appendix 4 Influence of moderators on the relationship between age and web-based health information quality.

Multimedia Appendix 5 Influence of moderators on the relationship between source-related factors and web-based health information quality.

Multimedia Appendix 6 Influence of moderators on the relationship between content-related factors and web-based health information quality.

Multimedia Appendix 7 Influence of moderators on the relationship between design-related factors and web-based health information quality.

Multimedia Appendix 8 Influence of moderators on the relationship between web-based health information quality and cognitive appraisals.

Multimedia Appendix 9 Influence of moderators on the relationship between web-based health information quality and behavioral intentions.

Multimedia Appendix 10 Influence of moderators on the relationship between web-based health information quality and behavior.

## Abbreviations

IQ: information quality
