# Supplementary material for: Consumers’ Evaluation of Web-Based Health Information Quality: Meta-analysis
Source: J Med Internet Res. 2022 Apr 28;24(4):e36463. doi: 10.2196/36463 (PMC9100526; doi:10.2196/36463)
Supplement: Multimedia Appendix 1 [file jmir_v24i4e36463_app1.docx]

**Multimedia Appendix 1. List of studies included in sample**

| **Study ID** | **Author(s)** | **Publication Outlet** | **Volume** | **Issue** | **Year** | **Sample Country** | **Study Method** | **Stimulus Type** | **Technology Context** | **Sample Clinical Status** | **Sample Type** | **Sample Size** | **Focal Variable** |
| --- | --- | --- | --- | --- | --- | --- | --- | --- | --- | --- | --- | --- | --- |
| 1 | Alkhalaf | Dissertation |  |  | 2013 | USA | Survey | General | Non-social media | Regular | Student | 323 | Credibility |
| 2 | Anderson | *Social Science Computer* | 22 | 2 | 2004 | USA | Survey | General | Non-social media | Regular | Non-student | 186 | Reliability |
| 3 | Anderson | *PloS One* | 13 | 10 | 2018 | Unknown/multiple countries | Survey | General | Non-social media | Regular | Non-student | 1,330 | Trust |
| 4 | Bao, Hoque, and Wang | *International Journal of Medical Informatics* | 102 |  | 2017 | China | Survey | General | Non-social media | Regular | Non-student | 218 | Trust |
| 5 | Barnes, Penrod, Neiger, Merrill, Thackeray, Eggett, and Thomas | *Journal of Health Psychology* | 8 | 1 | 2003 | USA | Survey | Specific | Non-social media | Regular | Non-student | 578 | Quality |
| 6 | Bates, Romina, Ahmed, and Hopson | *Medical Informatics and the Internet in Medicine* | 31 | 1 | 2006 | USA | Experiment | Specific | Non-social media | Regular | Non-student | 178 | Quality |
| 7 | Bates, Romina, Ahmed, and Hopson | *Medical Informatics and the Internet in Medicine* | 31 | 1 | 2006 | USA | Experiment | Specific | Non-social media | Regular | Non-student | 174 | Quality |
| 8 | Bates, Romina, Ahmed, and Hopson | *Medical Informatics and the Internet in Medicine* | 31 | 1 | 2006 | USA | Experiment | Specific | Non-social media | Regular | Non-student | 167 | Quality |
| 9 | Berger, Wagner, and Baker | *Social Science & Medicine* | 61 | 8 | 2005 | USA | Survey | General | Non-social media | Patient | Non-student | 2,115 | Trust |
| 10 | Bliemel | Dissertation |  |  | 2006 | Canada | Experiment | Specific | Non-social media | Regular | Non-student | 139 | Quality |
| 11 | Bode and Vraga | *Health Communication* | 33 | 9 | 2018 | USA | Experiment | Specific | Non-social media | Regular | Student | 136 | Credibility |
| 12 | Borah and Xiao | *Journal of Health Communication* | 23 | 4 | 2018 | USA | Experiment | Specific | Social media | Regular | Student | 340 | Credibility |
| 13 | Borah and Xiao | *Journal of Health Communication* | 23 | 4 | 2018 | USA | Experiment | Specific | Social media | Regular | Student | 552 | Credibility |
| 14 | Brown-Johnson, Boeckman, White, Burbank, Paulson, and Beebe | *JMIR Public Health and Surveillance* | 4 | 1 | 2018 | USA | Survey | General | Non-social media | Regular | Non-student | 1,001 | Trust |
| 15 | Cain, Armstrong, and Hou | *Online Journal of Communication and Media Technologies* | 10 | 2 | 2020 | USA | Survey | General | Non-social media | Regular | Student | 569 | Credibility |
| 16 | Cao, Zhang, Xu, and Wang | *Health Communication* | 31 | 9 | 2016 | China | Survey | General | Non-social media | Regular | Non-student | 393 | Credibility |
| 17 | Carpenter, DeVellis, Hogan, Fisher, DeVellis, and Jordan | *Journal of Health Communication* | 16 | 6 | 2011 | USA | Survey | General | Non-social media | Patient | Non-student | 230 | Credibility |
| 18 | Cash, Desbrow, Leveritt, and Ball | *Health Expectations* | 18 | 6 | 2015 | Australia | Survey | General | Non-social media | Regular | Non-student | 97 | Trust |
| 19 | Chen, Hay, Waters, Kiviniemi, Biddle, Schofield, Li, Kaphingst, and Orom | *Journal of Health Communication* | 23 | 8 | 2018 | USA | Survey | General | Non-social media | Regular | Non-student | 600 | Trust |
| 20 | Chen, Zheng, Liang, Xie, and Gu | *International Journal of Environmental Research and Public Health* | 17 | 13 | 2020 | China | Survey | General | Non-social media | Regular | Student | 491 | Quality |
| 21 | Cho, Lee, and Quinlan | *Journal of American College Health* | 63 | 4 | 2015 | USA | Survey | General | Non-social media | Regular | Student | 408 | Credibility |
| 22 | Choung, Lee, Jo, Shim, Lee, and Jung | *Journal of Korean Medical Science* | 32 | 7 | 2017 | South Korea | Survey | General | Non-social media | Regular | Non-student | 164 | Quality |
| 23 | Chua, Banerjee, Guan, Xian, and Peng | *SAI Computing Conference* |  |  | 2016 | Unknown/multiple countries | Experiment | Specific | Non-social media | Regular | Non-student | 110 | Trust |
| 24 | Chuang | *IEEE/WIC/ACM International Conference on Web Intelligence and Intelligent Agent Technology* |  |  | 2015 | Unknown/multiple countries | Experiment | Specific | Non-social media | Regular | Non-student | 482 | Credibility |
| 25 | Corritore, Wiedenbeck, Kracher, and Marble | *International Journal of Technology and Human Interaction* | 8 | 4 | 2012 | USA | Experiment | Specific | Non-social media | Regular | Student | 176 | Credibility |
| 26 | Cudmore, Bobrowski, and Kiguradze | *Journal of Consumer Marketing* | 28 | 4 | 2011 | USA | Experiment | Specific | Non-social media | Regular | Student | 192 | Quality |
| 27 | Deng, Liu, and Hinz | *Information Technology & People* | 28 | 2 | 2015 | China | Survey | General | Non-social media | Patient | Non-student | 259 | Quality |
| 28 | Diviani and Meppelink | *Computers in Human Behavior* | 71 |  | 2017 | USA | Experiment | Specific | Non-social media | Regular | Non-student | 403 | Credibility |
| 29 | Dutta, Pfister, and Kosmoski | *Journal of Computer-Mediated Communication* | 15 | 4 | 2010 | USA | Survey | Specific | Non-social media | Regular | Non-student | 118 | Quality |
| 30 | Dutta-Bergman | *Journal of Medical Internet Research* | 5 | 3 | 2003 | USA | Survey | General | Non-social media | Regular | Non-student | 2,636 | Credibility |
| 31 | Dutta-Bergman | *Journal of Communication* | 54 | 2 | 2004 | USA | Experiment | Specific | Non-social media | Regular | Student | 246 | Credibility |
| 32 | Eastin | *Journal of Computer-Mediated Communication* | 6 | 4 | 2001 | USA | Experiment | Specific | Non-social media | Regular | Student | 125 | Credibility |
| 33 | Embacher, McGloin, and Richards | *Western Journal of Communication* | 82 | 4 | 2018 | USA | Experiment | Specific | Social media | Regular | Student | 120 | Credibility |
| 34 | Escoffery | *Telemedicine and e-Health* | 24 | 5 | 2018 | USA | Survey | General | Non-social media | Regular | Non-student | 400 | Trust |
| 35 | Fan and Lederman | *European Journal of Information Systems* | 27 | 1 | 2018 | Australia | Survey | Specific | Social media | Regular | Non-student | 320 | Credibility |
| 36 | Fan, Huang, and Li | *Americas Conference on Information Systems* |  |  | 2019 | China | Experiment | Specific | Non-social media | Regular | Student | 120 | Quality |
| 37 | Fan, Zeng, and Shao | *Wuhan International Conference on e-Business* |  |  | 2017 | China | Experiment | Specific | Social media | Regular | Student | 80 | Credibility |
| 38 | Feng and Yang | *China Media Research* | 3 | 3 | 2007 | USA | Survey | General | Non-social media | Regular | Non-student | 5,480 | Credibility |
| 39 | Freeman and Spyridakis | *IEEE Transactions on Professional Communication* | 52 | 2 | 2009 | USA | Experiment | Specific | Non-social media | Regular | Student | 188 | Credibility |
| 40 | Furtado, Kaphingst, Perkins, and Politi | *Journal of Health Communication* | 21 | 2 | 2016 | USA | Survey | General | Non-social media | Regular | Non-student | 309 | Trust |
| 41 | Guo, Chen, Zhang, Ju, and Wang | *JMIR mHealth and uHealth* | 8 | 4 | 2020 | China | Survey | General | Non-social media | Regular | Non-student | 255 | Quality |
| 42 | Halkias, Harkiolakis, Thurman, and Caracatsanis | *Telemedicine and e-Health* | 14 | 3 | 2008 | Greece | Survey | General | Non-social media | Regular | Non-student | 70 | Trust |
| 43 | Harris, Sillence, and Briggs | *Journal of Medical Internet Research* | 13 | 3 | 2011 | Unknown/multiple countries | Survey | General | Non-social media | Regular | Non-student | 1,902 | Quality |
| 44 | Hether, Murphy, and Valente | *Journal of Health Communication* | 19 | 12 | 2014 | USA | Survey | Specific | Social media | Patient | Non-student | 114 | Trust |
| 45 | Hocevar | Dissertation |  |  | 2017 | Unknown/multiple countries | Experiment | Specific | Non-social media | Regular | Non-student | 479 | Credibility |
| 46 | Hocevar | Dissertation |  |  | 2017 | Unknown/multiple countries | Experiment | Specific | Non-social media | Regular | Non-student | 490 | Credibility |
| 47 | Hong | *International Communication Association* |  |  | 2003 | USA | Experiment | Specific | Non-social media | Regular | Student | 84 | Credibility |
| 48 | Jiang, Wu, Wang, and Chen | *International Conference on Information* |  |  | 2020 | China | Experiment | Specific | Non-social media | Regular | Student | 56 | Credibility |
| 49 | Jin, Phua, and Lee | *Computers in Human Behavior* | 46 |  | 2015 | USA | Experiment | Specific | Social media | Regular | Student | 102 | Trust |
| 50 | Jin, Phua, and Lee | *Computers in Human Behavior* | 46 |  | 2015 | USA | Experiment | Specific | Social media | Regular | Student | 294 | Trust |
| 51 | Johnson | *Ohio Communication Journal* | 57 |  | 2019 | USA | Survey | General | Non-social media | Patient | Non-student | 1,106 | Trust |
| 52 | Jucks and Thon | *Computers in Human Behavior* | 70 |  | 2017 | Germany | Experiment | Specific | Social media | Regular | Non-student | 78 | Credibility |
| 53 | Jung, Chung, and Rhee | *Health Communication* | 33 | 8 | 2018 | USA | Experiment | Specific | Non-social media | Regular | Non-student | 189 | Credibility |
| 54 | Jung, Walsh-Childers, and Kim | *Computers in Human Behavior* | 58 |  | 2016 | USA | Survey | Specific | Non-social media | Regular | Student | 575 | Credibility |
| 55 | Kalichman, Cherry, Cain, Weinhardt, Benotsch, Pope, and Kalichman | *Health Psychology* | 25 | 2 | 2006 | USA | Survey | Specific | Non-social media | Patient | Non-student | 419 | Quality |
| 56 | Kavathe | Dissertation |  |  | 2009 | USA | Survey | General | Non-social media | Regular | Non-student | 521 | Credibility |
| 57 | Khosrowjerdi | *Journal of Librarianship and Information Science* | 52 | 2 | 2020 | USA | Survey | General | Non-social media | Regular | Student | 171 | Credibility |
| 58 | Kim and Sundar | *Proceedings of the SIGCHI Conference on Human Factors in Computing Systems* |  |  | 2011 | USA | Experiment | Specific | Social media | Regular |  | 99 | Credibility |
| 59 | Kim, Han, Yoo, and Yun | *Telemedicine and e-Health* | 18 | 9 | 2012 | South Korea | Survey | General | Non-social media | Regular | Non-student | 449 | Credibility |
| 60 | König and Jucks | *International Journal of Educational Technology in Higher Education* | 16 | 1 | 2019 | Germany | Experiment | Specific | Non-social media | Regular | Student | 189 | Credibility |
| 61 | Koo, Wati, Park, and Lim | *Journal of Medical Internet Research* | 13 | 4 | 2011 | South Korea | Survey | General | Non-social media | Regular | Non-student | 198 | Quality |
| 62 | Kwon, Kye, Park, Oh, and Park | *Epidemiology and Health* | 37 |  | 2015 | South Korea | Survey | General | Non-social media | Regular | Non-student | 1,300 | Trust |
| 63 | Lazard and Mackert | *International Journal of Medical Informatics* | 83 | 10 | 2014 | USA | Experiment | Specific | Non-social media | Regular | Student | 231 | Quality |
| 64 | Lederman, Fan, Smith, and Chang | *Health Policy and Technology* | 3 | 1 | 2014 | USA | Survey | General | Social media | Regular | Non-student | 140 | Credibility |
| 65 | Lee and Chae | *Health Communication* | 31 | 11 | 2016 | USA | Survey | General | Non-social media | Regular | Non-student | 3,959 | Trust |
| 66 | Lee, Choi, and Noh | *Journal of Health Communication* | 21 | 11 | 2016 | South Korea | Survey | General | Non-social media | Regular | Non-student | 1,200 | Trust |
| 67 | Lee, Dutta, Lin, Luk, and Kaur-Gill | *Journal of Health Communication* | 23 | 3 | 2018 | Singapore | Survey | General | Non-social media | Regular | Non-student | 273 | Trust |
| 68 | Lee, Dutta, Lin, Luk, and Kaur-Gill | *Journal of Health Communication* | 23 | 3 | 2018 | Singapore | Survey | General | Non-social media | Regular | Non-student | 324 | Trust |
| 69 | Lee, Park, and Widdows | *Journal of Health Communication* | 14 | 2 | 2009 | USA | Survey | General | Non-social media | Regular | Non-student | 252 | Credibility |
| 70 | Lemire, Paré, Sicotte, and Harvey | *International Journal of Medical Informatics* | 77 | 11 | 2008 | Canada | Survey | Specific | Non-social media | Regular | Non-student | 2,923 | Trust |
| 71 | Leung | *CyberPsychology & Behavior* | 11 | 5 | 2008 | Hong Kong | Survey | General | Non-social media | Regular | Non-student | 1,076 | Quality |
| 72 | Li and Wang | *Journal of Organizational and End User Computing* | 30 | 1 | 2018 | Italy | Survey | General | Social media | Regular | Non-student | 137 | Trust |
| 73 | Liang and Xue | *International Conference on Information Systems* |  |  | 2013 | Unknown/multiple countries | Survey | General | Non-social media | Patient | Non-student | 243 | Quality |
| 74 | Lieberman, Lingsweiler, Yao, and Chesler | *International Communication Association* |  |  | 2003 | Unknown/multiple countries | Experiment | Specific | Non-social media | Regular | Student | 117 | Quality |
| 75 | Lim and Kim | *Informatics for Health and Social Care* |  |  | 2012 | South Korea | Survey | General | Non-social media | Regular | Student | 374 | Trust |
| 76 | Lin, Zhang, Song, and Omori | *Computers in Human Behavior* | 56 |  | 2016 | USA | Survey | General | Social media | Regular | Student | 234 | Trust |
| 77 | Lin, Zhang, Song, and Omori | *Computers in Human Behavior* | 56 |  | 2016 | Hong Kong | Survey | General | Social media | Regular | Student | 249 | Trust |
| 78 | Lin, Zhang, Song, and Omori | *Computers in Human Behavior* | 56 |  | 2016 | South Korea | Survey | General | Social media | Regular | Student | 151 | Trust |
| 79 | Litchman | Dissertation |  |  | 2015 | USA | Survey | General | Social media | Patient | Non-student | 178 | Credibility |
| 80 | Liu, Zhang, and Lu | *Telemedicine and e-Health* | 25 | 11 | 2019 | China | Survey | General | Non-social media | Regular | Non-student | 336 | Quality |
| 81 | Lu, Zhang, Wu, and Shang | *International Conference on Information Communication and Management* |  |  | 2018 | China | Survey | General | Non-social media | Regular | Non-student | 401 | Quality |
| 82 | Machackova and Smahel | *Telematics and Informatics* | 35 | 5 | 2018 | Czech Republic | Survey | Specific | Non-social media | Regular | Non-student | 659 | Credibility |
| 83 | Maguire, Reay, Looi, Cubis, Byrne, and Raphael | *Australian & New Zealand Journal of Psychiatry* | 45 | 6 | 2011 | Australia | Survey | General | Non-social media | Patient | Non-student | 238 | Trust |
| 84 | Maguire, Reay, Looi, Cubis, Byrne, and Raphael | *Australian & New Zealand Journal of Psychiatry* | 45 | 6 | 2011 | Australia | Survey | General | Non-social media | Patient | Non-student | 71 | Trust |
| 85 | Mano | *Health Expectations* | 18 | 6 | 2015 | Israel | Survey | General | Non-social media | Regular | Non-student | 1,406 | Trust |
| 86 | Marrie, Salter, Tyry, Fox, and Cutter | *Journal of Medical Internet Research* | 15 | 4 | 2013 | USA | Survey | General | Non-social media | Patient | Non-student | 8,586 | Trust |
| 87 | McKinley and Ruppel | *Computers in Human Behavior* | 34 |  | 2014 | USA | Survey | General | Non-social media | Regular | Student | 350 | Trust |
| 88 | Medlock, Eslami, Askari, Arts, Sent, de Rooij, and Abu-Hanna | *Journal of Medical Internet Research* | 17 | 1 | 2015 | Netherlands | Survey | General | Non-social media | Regular | Non-student | 118 | Trust |
| 89 | Meppelink, Smit, Fransen, and Diviani | *Journal of Health Communication* | 24 | 2 | 2019 | USA | Survey | General | Non-social media | Regular | Non-student | 480 | Credibility |
| 90 | Mun, Yoon, Davis, and Lee | *Decision Support Systems* | 55 | 1 | 2013 | South Korea | Experiment | Specific | Non-social media | Regular | Non-student | 300 | Quality |
| 91 | Neter and Brainin | *Journal of Medical Internet Research* | 14 | 1 | 2012 | Israel | Survey | General | Non-social media | Regular | Non-student | 1,289 | Credibility |
| 92 | Neubaum and Krämer | *Health Communication* | 30 | 9 | 2015 | Germany | Experiment | Specific | Social media | Regular | Non-student | 261 | Credibility |
| 93 | Neumark, Lopez-Quintero, Feldman, Allen, and Shtarkshall | *Journal of Health Communication* | 18 | 9 | 2013 | Israel | Survey | General | Non-social media | Regular | Student | 3,449 | Trust |
| 94 | Nunn, Crutzen, Haag, Chabot, Carson, Ogilvie, Shoveller, and Gilbert | *JMIR Public Health and Surveillance* | 3 | 4 | 2017 | Canada | Survey | Specific | Non-social media | Regular | Non-student | 173 | Trust |
| 95 | Oh, Jun, Zhao, Kreps, and Lee | *Journal of Health Communication* | 20 | 10 | 2015 | USA | Survey | General | Non-social media | Regular | Non-student | 152 | Trust |
| 96 | Paige, Krieger, and Stellefson | *Journal of Health Communication* | 22 | 1 | 2017 | USA | Survey | General | Social media | Regular | Non-student | 811 | Trust |
| 97 | Pan | *International Communication Association* |  |  | 2006 | USA | Experiment | Specific | Non-social media | Regular | Student | 232 | Credibility |
| 98 | Park and Go | *Health Marketing Quarterly* | 33 | 4 | 2016 | USA | Experiment | Specific | Non-social media | Regular | Student | 71 | Quality |
| 99 | Park, Chung, and Yoo | *Journal of Retailing and Consumer Services* | 16 | 2 | 2009 | Unknown/multiple countries | Survey | General | Non-social media | Regular | Non-student | 151 | Credibility |
| 100 | Park, Chung, and Yoo | *Journal of Retailing and Consumer Services* | 16 | 2 | 2009 | Unknown/multiple countries | Survey | General | Non-social media | Regular | Non-student | 109 | Credibility |
| 101 | Peng and Logan | *International Communication Association* |  |  | 2005 | USA | Survey | Specific | Non-social media | Regular | Non-student | 374 | Quality |
| 102 | Phua and Tinkham | *Journal of Health Communication* | 21 | 3 | 2016 | USA | Experiment | Specific | Non-social media | Regular | Non-student | 200 | Credibility |
| 103 | Purcarea, Gheorghe, Petrescu | *Journal of Medicine and Life* | 6 | 3 | 2013 | Romania | Survey | General | Non-social media | Regular | Non-student | 127 | Credibility |
| 104 | Qiu, Ren, Liu, Yin, and Ren | *BMJ Open* | 9 | 5 | 2019 | China | Survey | General | Non-social media | Regular | Non-student | 652 | Trust |
| 105 | Rains | *Journal of Applied Communication Research* | 35 | 2 | 2007 | USA | Experiment | Specific | Non-social media | Regular | Non-student | 255 | Credibility |
| 106 | Rains | *Western Journal of Communication* | 72 | 1 | 2008 | USA | Survey | General | Non-social media | Regular | Student | 151 | Quality |
| 107 | Rains and Karmikel | *Computers in Human Behavior* | 25 | 2 | 2009 | USA | Experiment | Specific | Non-social media | Regular | Student | 86 | Credibility |
| 108 | Ray, Sewell, Gilbert, and Roberts | *Journal of Health Politics, Policy and Law* | 42 | 5 | 2017 | USA | Survey | General | Non-social media | Regular | Non-student | 1,036 | Trust |
| 109 | Record, Silberman, Santiago, and Ham | *Journal of Health Communication* | 23 | 5 | 2018 | Unknown/multiple countries | Survey | Specific | Social media | Regular | Non-student | 389 | Credibility |
| 110 | Robertson-Lang, Major, and Hemming | *Canadian Journal on Aging/La Revue Canadienne du Vieillissement* | 30 | 4 | 2011 | Canada | Experiment | Specific | Non-social media | Regular | Non-student | 83 | Quality |
| 111 | Robins, Holmes, and Stansbury | *Journal of the American Society for Information Science and Technology* | 61 | 1 | 2010 | USA | Experiment | Specific | Non-social media | Regular | Non-student | 34 | Credibility |
| 112 | Rutten, Blake, Hesse, and Ackerson | *Journal of Cancer Education* | 26 | 3 | 2011 | USA | Survey | General | Non-social media | Patient | Non-student | 5,586 | Trust |
| 113 | Sauls | Dissertation |  |  | 2018 | Unknown/multiple countries | Experiment | Specific | Social media | Regular | Non-student | 362 | Credibility |
| 114 | Sbaffi and Zhao | *Journal of the Association for Information Science and Technology* | 71 | 2 | 2020 | UK | Experiment | Specific | Non-social media | Regular | Student | 291 | Credibility |
| 115 | Schaefer | *National Communication Association* |  |  | 2008 | USA | Experiment | Specific | Non-social media | Regular | Student | 124 | Credibility |
| 116 | Schweiger and Cress | *Journal of Medical Internet Research* | 21 | 4 | 2019 | Germany | Experiment | Specific | Non-social media | Regular | Non-student | 226 | Credibility |
| 117 | Seckin | *Social Science & Medicine* | 247 |  | 2020 | USA | Survey | General | Non-social media | Regular | Non-student | 710 | Trust |
| 118 | Selsky, Luta, Noone, Huerta, and Mandelblatt | *Journal of Health Communication* | 18 | 1 | 2013 | USA | Survey | General | Non-social media | Patient | Non-student | 1,273 | Trust |
| 119 | Shakir, Wong, Abdullah, and Adam | *Sexual Health* | 16 | 2 | 2019 | Malaysia | Survey | General | Non-social media | Regular | Student | 1,530 | Quality |
| 120 | Shen, Xu, and Wang | *Journal of Health Communication* | 24 | 2 | 2019 | USA | Survey | General | Non-social media | Regular | Non-student | 2,293 | Trust |
| 121 | Sheng and Simpson | *Health Marketing Quarterly* | 32 | 1 | 2015 | USA | Survey | General | Non-social media | Regular | Non-student | 832 | Trust |
| 122 | Shim and Jo | *International Journal of Medical Informatics* | 137 |  | 2020 | South Korea | Survey | Specific | Non-social media | Regular | Non-student | 506 | Quality |
| 123 | Shon, Marshall, and Musen | *American Medical Informatics Association* |  |  | 2000 | USA | Experiment | Specific | Non-social media | Regular | Non-student | 137 | Credibility |
| 124 | Sillence, Blythe, Briggs, and Moss | *Journal of Medical Internet Research* | 21 | 11 | 2019 | USA | Survey | Specific | Non-social media | Regular | Non-student | 625 | Trust |
| 125 | Sillence, Blythe, Briggs, and Moss | *Journal of Medical Internet Research* | 21 | 11 | 2019 | UK | Survey | Specific | Non-social media | Regular | Non-student | 498 | Trust |
| 126 | Song, Omori, Kim, Tenzek, Hawkins, Lin, Kim, and Jung | *Journal of Medical Internet Research* | 18 | 3 | 2016 | Unknown/multiple countries | Survey | General | Social media | Regular | Student | 826 | Trust |
| 127 | Song, Zhao, Song, and Zhu | *IEEE International Conference on Healthcare Informatics* | 56 | 1 | 2019 | China | Experiment | Specific | Non-social media | Regular | Non-student | 252 | Credibility |
| 128 | Song, Zhao, Song, and Zhu | *Association for Information Science and Technology* |  |  | 2019 | China | Experiment | Specific | Non-social media | Regular | Non-student | 218 | Credibility |
| 129 | Spence, Lachlan, Westerman, and Spates | *Howard Journal of Communications* | 24 | 1 | 2013 | USA | Experiment | Specific | Social media | Regular | Student | 200 | Credibility |
| 130 | Syn and Kim | *American Society for Information Science and Technology* |  |  | 2013 | USA | Survey | Specific | Social media | Regular | Student | 200 | Credibility |
| 131 | Tao, Yuan, Shao, Li, Zhou, and Qu | *CIN: Computers, Informatics, Nursing* | 36 | 11 | 2018 | China | Survey | Specific | Non-social media | Regular | Student | 201 | Credibility |
| 132 | Tedesco and Holloway | *Studies in Communication Sciences* | 5 | 2 | 2005 | USA | Experiment | Specific | Non-social media | Regular | Student | 212 | Credibility |
| 133 | Thombre | Dissertation |  |  | 2004 | USA | Experiment | Specific | Non-social media | Regular | Student | 300 | Credibility |
| 134 | Thomm and Bromme | *Science Education* | 96 | 2 | 2012 | Germany | Experiment | Specific | Non-social media | Regular | Student | 78 | Credibility |
| 135 | Thompson | *National Communication Association* |  |  | 2007 | Unknown/multiple countries | Survey | Specific | Social media | Regular | Non-student | 65 | Trust |
| 136 | Thon and Jucks | *Health Communication* | 32 | 7 | 2017 | Germany | Experiment | Specific | Non-social media | Regular | Student | 127 | Credibility |
| 137 | Van de Belt, Engelen, Berben, Teerenstra, Samsom, and Schoonhoven | *Journal of Medical Internet Research* | 15 | 10 | 2013 | Netherlands | Survey | General | Non-social media | Regular | Non-student | 635 | Reliability |
| 138 | Van de Vord | *International Communication Association* |  |  | 2007 | USA | Survey | General | Non-social media | Regular | Student | 202 | Credibility |
| 139 | Walther, Jang, and Hanna Edwards | *Health Communication* | 33 | 1 | 2018 | USA | Experiment | Specific | Social media | Regular |  | 354 | Quality |
| 140 | Wang, Walther, Pingree, and Hawkins | *Health Communication* | 23 | 4 | 2008 | USA | Experiment | Specific | Non-social media | Regular | Non-student | 97 | Credibility |
| 141 | Williams, Ames, and Lawson | *Journal of Communication in Healthcare* | 12 | 2 | 2019 | Australia | Survey | General | Non-social media | Regular | Non-student | 1,013 | Trust |
| 142 | Yang and Li | *Computers in Human Behavior* | 64 |  | 2016 | USA | Experiment | Specific | Social media | Regular | Non-student | 224 | Credibility |
| 143 | Ye | *Journal of Health Communication* | 15 | Suppl. 3 | 2010 | USA | Survey | General | Non-social media | Regular | Non-student | 7,674 | Trust |
| 144 | Yun and Park | *Journal of Clinical Nursing* | 19 | 19-20 | 2010 | South Korea | Survey | General | Non-social media | Regular | Non-student | 212 | Credibility |
| 145 | Zimmerman and Jucks | *Interactive Journal of Medical Research* | 7 | 2 | 2018 | Germany | Experiment | Specific | Non-social media | Regular | Non-student | 151 | Credibility |
| 146 | Zimmermann and Jucks | *Journal of Medical Internet Research* | 20 | 1 | 2018 | Germany | Experiment | Specific | Non-social media | Regular | Non-student | 98 | Credibility |
| 147 | Zulman, Kirch, Zheng, and An | *Journal of Medical Internet Research* | 13 | 1 | 2011 | USA | Survey | General | Non-social media | Regular | Non-student | 1,450 | Trust |

**References**

[1] Alkhalaf, A. A. (2013). *Exploring university students’ online information seeking about prescription medications*. ProQuest. The Florida State University.

[2] Anderson, J. G. (2004). Consumers of e-health: patterns of use and barriers. *Social Science Computer Review*, *22*(2), 242-248.

[3] Anderson, A. (2018). Online health information and public knowledge, attitudes, and behaviours regarding antibiotics in the UK: Multiple regression analysis of Wellcome Monitor and Eurobarometer Data. *PloS One*, *13*(10), e0204878.

[4] Bao, Y., Hoque, R., & Wang, S. (2017). Investigating the determinants of Chinese adult children’s intention to use online health information for their aged parents. *International Journal of Medical Informatics*, *102*, 12-20.

[5] Barnes, M. D., Penrod, C., Neiger, B. L., Merrill, R. M., Thackeray, R., Eggett, D. L., & Thomas, E. (2003). Measuring the relevance of evaluation criteria among health information seekers on the Internet. *Journal of Health Psychology*, *8*(1), 71-82.

[6-8] Bates, B. R., Romina, S., Ahmed, R., & Hopson, D. (2006). The effect of source credibility on consumers' perceptions of the quality of health information on the Internet. *Medical Informatics and the Internet in Medicine*, *31*(1), 45-52.

[9] Berger, M., Wagner, T. H., & Baker, L. C. (2005). Internet use and stigmatized illness. *Social Science & Medicine*, *61*(8), 1821-1827.

[10] Bliemel, M. (2006). Consumer satisfaction with online health information: A theoretical model and an empirical study. McMaster University. Accessed on Feb. 19, 2022 at: <https://macsphere.mcmaster.ca/bitstream/11375/20114/1/Bliemel_Michael_2006_04_phd.pdf>

[11] Bode, L., & Vraga, E. K. (2018). See something, say something: Correction of global health misinformation on social media. *Health Communication*, *33*(9), 1131-1140.

[12-13] Borah, P., & Xiao, X. (2018). The importance of ‘likes’: The interplay of message framing, source, and social endorsement on credibility perceptions of health information on Facebook. *Journal of Health Communication*, *23*(4), 399-411.

[14] Brown-Johnson, C. G., Boeckman, L. M., White, A. H., Burbank, A. D., Paulson, S., & Beebe, L. A. (2018). Trust in health information sources: survey analysis of variation by sociodemographic and tobacco use status in Oklahoma. *JMIR Public Health and Surveillance*, *4*(1), e6260.

[15] Cain, J. A., Armstrong, C., & Hou, J. (2020). Somebody Google a doctor! Urgent health information seeking habits of young adults. *Online Journal of Communication and Media Technologies*, *10*(2), e202006.

[16] Cao, W., Zhang, X., Xu, K., & Wang, Y. (2016). Modeling online health information-seeking behavior in China: The roles of source characteristics, reward assessment, and internet self-efficacy. *Health Communication*, *31*(9), 1105-1114.

[17] Carpenter, D. M., DeVellis, R. F., Hogan, S. L., Fisher, E. B., DeVellis, B. M., & Jordan, J. M. (2011). Use and perceived credibility of medication information sources for patients with a rare illness: differences by gender. *Journal of Health Communication*, *16*(6), 629-642.

[18] Cash, T., Desbrow, B., Leveritt, M., & Ball, L. (2015). Utilization and preference of nutrition information sources in Australia. *Health Expectations*, *18*(6), 2288-2295.

[19] Chen, X., Hay, J. L., Waters, E. A., Kiviniemi, M. T., Biddle, C., Schofield, E., ... & Orom, H. (2018). Health literacy and use and trust in health information. *Journal of Health Communication*, *23*(8), 724-734.

[20] Chen, W., Zheng, Q., Liang, C., Xie, Y., & Gu, D. (2020). Factors Influencing College Students’ Mental Health Promotion: The Mediating Effect of Online Mental Health Information Seeking. *International Journal of Environmental Research and Public Health*, *17*(13), 4783.

[21] Cho, J., Lee, H. E., & Quinlan, M. (2015). Complementary relationships between traditional media and health apps among American college students. *Journal of American College Health*, *63*(4), 248-257.

[22] Choung, J. T., Lee, Y. S., Jo, H. S., Shim, M., Lee, H. J., & Jung, S. M. (2017). What factors impact consumer perception of the effectiveness of health information sites? An investigation of the Korean National Health Information Portal. *Journal of Korean Medical Science*, *32*(7), 1077-1082.

[23] Chua, A. Y., Banerjee, S., Guan, A. H., Xian, L. J., & Peng, P. (2016, July). Intention to trust and share health-related online rumors: Studying the role of risk propensity. *2016 SAI Computing Conference (SAI)* (pp. 1136-1139). IEEE.

[24] Chuang, C.-P. (2015). Effects of Audience Characteristics and Sources of Information on Perceived Credibility of Web Information. *2015 IEEE/WIC/ACM International Conference on Web Intelligence and Intelligent Agent Technology* *(WI-IAT)*, 110–113. <https://doi.org/10.1109/WI-IAT.2015.261>

[25] Corritore, C. L., Wiedenbeck, S., Kracher, B., & Marble, R. P. (2012). Online trust and health information websites. *International Journal of Technology and Human Interaction (IJTHI)*, *8*(4), 92-115.

[26] Cudmore, B. A., Bobrowski, P. E., & Kiguradze, T. (2011). Encouraging consumer searching behavior on healthcare web sites. *Journal of Consumer Marketing*, *28*(4), 290-299.

[27] Deng, Z., Liu, S., & Hinz, O. (2015). The health information seeking and usage behavior intention of Chinese consumers through mobile phones. *Information Technology & People*, *28*(2), 405-423.

[28] Diviani, N., & Meppelink, C. S. (2017). The impact of recommendations and warnings on the quality evaluation of health websites: An online experiment. *Computers in Human Behavior*, *71*, 122-129.

[29] Dutta, M. J., Pfister, R., & Kosmoski, C. (2010). Consumer evaluation of genetic information online: The role of quality on attitude and behavioral intentions. *Journal of Computer-Mediated Communication*, *15*(4), 592-605.

[30] Dutta-Bergman, M. (2003). Trusted online sources of health information: differences in demographics, health beliefs, and health-information orientation. *Journal of Medical Internet Research*, *5*(3), e893.

[31] Dutta-Bergman, M. J. (2004). The impact of completeness and web use motivation on the credibility of e-health information. *Journal of Communication*, *54*(2), 253-269.

[32] Eastin, M. S. (2001). Credibility assessments of online health information: The effects of source expertise and knowledge of content. *Journal of Computer-Mediated Communication*, *6*(4), JCMC643.

[33] Embacher, K., McGloin, R., & Richards, K. (2018). When women give health advice online, do we listen? The effect of source sex on credibility and likelihood to use online health advice. *Western Journal of Communication*, *82*(4), 439-456.

[34] Escoffery, C. (2018). Gender similarities and differences for e-Health behaviors among US adults. *Telemedicine and e-Health*, *24*(5), 335-343.

[35] Fan, H., & Lederman, R. (2018). Online health communities: how do community members build the trust required to adopt information and form close relationships?. *European Journal of Information Systems*, *27*(1), 62-89.

[36] Fan, J., Huang, X., & Li, Y. (2019). The Effect of Features on Information Quality and Knowledge Acquisition in Online Health Communities.

[37] Fan, J., Zeng, Y., & Shao, M. (2017). How to Improve the Credibility and Interestingness of Social Media Healthcare Information?. *Wuhan International Conference on e-Business*. Association for Information Systems.

[38] Feng, H., & Yang, Y. (2007). A Model of Cancer-Related Health Information Seeking on the Internet. *China Media Research*, *3*(3).

[39] Freeman, K. S., & Spyridakis, J. H. (2009). Effect of contact information on the credibility of online health information. *IEEE Transactions on Professional Communication*, *52*(2), 152-166.

[40] Furtado, K. S., Kaphingst, K. A., Perkins, H., & Politi, M. C. (2016). Health Insurance Information-Seeking Behaviors Among the Uninsured. *Journal of Health Communication*, *21*(2), 148–158.

[41] Guo, X., Chen, S., Zhang, X., Ju, X., & Wang, X. (2020). Exploring patients' intentions for continuous usage of mHealth services: elaboration-likelihood perspective study. *JMIR mHealth and uHealth*, *8*(4), e17258.

[42] Halkias, D., Harkiolakis, N., Thurman, P., & Caracatsanis, S. (2008). Internet use for health-related purposes among Greek consumers. *Telemedicine and e-Health*, *14*(3), 255-260.

[43] Harris, P. R., Sillence, E., & Briggs, P. (2011). Perceived threat and corroboration: key factors that improve a predictive model of trust in internet-based health information and advice. *Journal of Medical Internet Research*, *13*(3), e1821.

[44] Hether, H. J., Murphy, S. T., & Valente, T. W. (2014). It's better to give than to receive: The role of social support, trust, and participation on health-related social networking sites. *Journal of Health Communication*, *19*(12), 1424-1439.

[45-46] Hocevar, K. P. (2017). *Help or Hurt? Why We Select and How We Process Online Social Information About Health.* ProQuest. University of California Santa Barbara.

[47] Hong, T. (2003). Information Seeking of Health-Related Web Sites. *International Communication Association 2003 Annual Meeting*, San Diego, CA, pp.1–43.

[48] Jiang, T., Wu, X., Wang, Y., & Chen, Y. (2020, March). The Effects of Message Framing on Online Health Headline Selection: A Mediation of Message Credibility. *International Conference on Information*. Springer, Cham. pp. 428-437.

[49-50] Jin, S. V., Phua, J., & Lee, K. M. (2015). Telling stories about breastfeeding through Facebook: The impact of user-generated content (UGC) on pro-breastfeeding attitudes. *Computers in Human Behavior*, *46*, 6-17.

[51] Johnson, N. L. (2019). Health Information-Seeking Behaviors and Disparities Among Patients with Type 2 Diabetes: Testing Predictors of the Frequency of HISB with Doctors and Online. *Ohio Communication Journal*, *57*, 75-90.

[52] Jucks, R., & Thon, F. M. (2017). Better to have many opinions than one from an expert? Social validation by one trustworthy source versus the masses in online health forums. *Computers in Human Behavior*, *70*, 375-381.

[53] Jung, W. S., Chung, M. Y., & Rhee, E. S. (2018). The effects of attractiveness and source expertise on online health sites. *Health Communication*, *33*(8), 962-971.

[54] Jung, E. H., Walsh-Childers, K., & Kim, H. S. (2016). Factors influencing the perceived credibility of diet-nutrition information web sites. *Computers in Human Behavior*, *58*, 37-47.

[55] Kalichman, S. C., Cherry, C., Cain, D., Weinhardt, L. S., Benotsch, E., Pope, H., & Kalichman, M. (2006). Health information on the Internet and people living with HIV/AIDS: information evaluation and coping styles. *Health Psychology*, *25*(2), 205-210.

[56] Kavathe, R. S. (2009). *Patterns of Access and Use of Online Health Information among Internet Users: A Case Study*. Bowling Green State University. <https://etd.ohiolink.edu/apexprod/rws_etd/send_file/send?accession=bgsu1250696451&disposition=inline>

[57] Khosrowjerdi, M. (2020). National culture and trust in online health information. *Journal of Librarianship and Information Science*, *52*(2), 509-528.

[58] Kim, H. S., & Sundar, S. S. (2011, May). Using interface cues in online health community boards to change impressions and encourage user contribution. *Proceedings of the SIGCHI Conference on Human Factors in Computing Systems* (pp. 599-608). ACM

[59] Kim, N. E., Han, S. S., Yoo, K. H., & Yun, E. K. (2012). The impact of user's perceived ability on online health information acceptance. *Telemedicine and e-Health*, *18*(9), 703-708.

[60] König, L., & Jucks, R. (2019). When do information seekers trust scientific information? Insights from recipients’ evaluations of online video lectures. *International Journal of Educational Technology in Higher Education*, *16*(1), 1-21.

[61] Koo, C., Wati, Y., Park, K., & Lim, M. K. (2011). Website quality, expectation, confirmation, and end user satisfaction: the knowledge-intensive website of the Korean National Cancer Information Center. *Journal of Medical Internet Research*, *13*(4), e1574.

[62] Kwon, J. H., Kye, S. Y., Park, E. Y., Oh, K. H., & Park, K. (2015). What predicts the trust of online health information?. *Epidemiology and health*, *37*, e2015030.

[63] Lazard, A., & Mackert, M. (2014). User evaluations of design complexity: The impact of visual perceptions for effective online health communication. *International Journal of Medical Informatics*, *83*(10), 726-735.

[64] Lederman, R., Fan, H., Smith, S., & Chang, S. (2014). Who can you trust? Credibility assessment in online health forums. *Health Policy and Technology*, *3*(1), 13-25.

[65] Lee, C. J., & Chae, J. (2016). An initial look at the associations of a variety of health-related online activities with cancer fatalism. *Health Communication*, *31*(11), 1375-1384.

[66] Lee, S. Y., Choi, J., & Noh, G. Y. (2016). Factors influencing health-related internet activities and their outcomes. *Journal of Health Communication*, *21*(11), 1179-1186.

[67-68] Lee, S. T., Dutta, M. J., Lin, J., Luk, P., & Kaur-Gill, S. (2018). Trust ecologies and channel complementarity for information seeking in cancer prevention. *Journal of Health Communication*, *23*(3), 254-263.

[69] Lee, Y. J., Park, J., & Widdows, R. (2009). Exploring antecedents of consumer satisfaction and repeated search behavior on e-health information. *Journal of Health Communication*, *14*(2), 160-173.

[70] Lemire, M., Paré, G., Sicotte, C., & Harvey, C. (2008). Determinants of Internet use as a preferred source of information on personal health. *International Journal of Medical Informatics*, *77*(11), 723-734.

[71] Leung, L. (2008). Internet embeddedness: links with online health information seeking, expectancy value/quality of health information websites, and Internet usage patterns. *CyberPsychology & Behavior*, *11*(5), 565-569.

[72] Li, Y., & Wang, X. (2018). Seeking health information on social media: a perspective of trust, self-determination, and social support. *Journal of Organizational and End User Computing (JOEUC)*, *30*(1), 1-22.

[73] Liang, H., & Xue, Y. (2013). Online health information use by disabled people: The moderating role of disability. *34^th^ International Conference on Information Systems*. pp. 1-16.

[74] Lieberman, D., Lingsweiler, R., Yao, M., & Chesler, Z. (2003). Effects of User Control and Perceived Message Tailoring on Responses to a Health Web Site. *International Communication Association*, pp. 1–16.

[75] Lim, S. H., & Kim, D. (2012). The role of trust in the use of health infomediaries among university students. *Informatics for Health and Social Care*, *37*(2), 92-105.

[76-78] Lin, W. Y., Zhang, X., Song, H., & Omori, K. (2016). Health information seeking in the Web 2.0 age: Trust in social media, uncertainty reduction, and self-disclosure. *Computers in Human Behavior*, *56*, 289-294.

[79] Litchman, M. L. (2015). *A multiple method analysis of peer health in the diabetes online community*. The University of Utah. Retrieved February 19, 2022, from <https://www.proquest.com/docview/1755696611/abstract/35D5821145CC4E23PQ/1>

[80] Liu, S., Zhang, R., & Lu, X. (2019). The Impact of Individuals' Attitudes Toward Health Websites on Their Perceived Quality of Health Information: An Empirical Study. *Telemedicine and e-Health*, *25*(11), 1099-1107.

[81] Lu, X., Zhang, R., Wu, W., & Shang, X. (2018, August). How does health website influence patient compliance: an empirical study. *Proceedings of the 8th International Conference on Information Communication and Management*, pp. 50-55.

[82] Machackova, H., & Smahel, D. (2018). The perceived importance of credibility cues for the assessment of the trustworthiness of online information by visitors of health-related websites: The role of individual factors. *Telematics and Informatics*, *35*(5), 1534-1541.

[83-84] Maguire, P. A., Reay, R. E., Looi, J. C., Cubis, J., Byrne, G. J., & Raphael, B. (2011). Neither the internist nor the Internet: use of and trust in health information sources by people with schizophrenia. *Australian & New Zealand Journal of Psychiatry*, *45*(6), 489-497.

[85] Mano, R. (2015). Online health information, situational effects and health changes among e‐patients in Israel: A ‘push/pull’perspective. *Health Expectations*, *18*(6), 2489-2500.

[86] Marrie, R. A., Salter, A. R., Tyry, T., Fox, R. J., & Cutter, G. R. (2013). Preferred sources of health information in persons with multiple sclerosis: degree of trust and information sought. *Journal of Medical Internet Research*, *15*(4), e2466.

[87] McKinley, C. J., & Ruppel, E. K. (2014). Exploring how perceived threat and self-efficacy contribute to college students’ use and perceptions of online mental health resources. *Computers in Human Behavior*, *34*, 101-109.

[88] Medlock, S., Eslami, S., Askari, M., Arts, D. L., Sent, D., De Rooij, S. E., & Abu-Hanna, A. (2015). Health information–seeking behavior of seniors who use the internet: a survey. *Journal of Medical Internet Research*, *17*(1), e3749.

[89] Meppelink, C. S., Smit, E. G., Fransen, M. L., & Diviani, N. (2019). “I was right about vaccination”: Confirmation bias and health literacy in online health information seeking. *Journal of Health Communication*, *24*(2), 129-140.

[90] Mun, Y. Y., Yoon, J. J., Davis, J. M., & Lee, T. (2013). Untangling the antecedents of initial trust in Web-based health information: The roles of argument quality, source expertise, and user perceptions of information quality and risk. *Decision Support Systems*, *55*(1), 284-295.

[91] Neter, E., & Brainin, E. (2012). eHealth literacy: extending the digital divide to the realm of health information. *Journal of Medical Internet Research*, *14*(1), e1619.

[92] Neubaum, G., & Krämer, N. C. (2015). Let’s blog about health! Exploring the persuasiveness of a personal HIV blog compared to an institutional HIV website. *Health Communication*, *30*(9), 872-883.

[93] Neumark, Y., Lopez-Quintero, C., Feldman, B. S., Hirsch Allen, A. J., & Shtarkshall, R. (2013). Online health information seeking among Jewish and Arab adolescents in Israel: Results from a national school survey. *Journal of Health Communication*, *18*(9), 1097-1115.

[94] Nunn, A., Crutzen, R., Haag, D., Chabot, C., Carson, A., Ogilvie, G., ... & Gilbert, M. (2017). Examining e-loyalty in a sexual health website: Cross-sectional study. *JMIR Public Health and Surveillance*, *3*(4), e5393.

[95] Oh, K. M., Jun, J., Zhao, X., Kreps, G. L., & Lee, E. E. (2015). Cancer information seeking behaviors of Korean American women: A mixed-methods study using surveys and focus group interviews. *Journal of Health Communication*, *20*(10), 1143-1154.

[96] Paige, S. R., Krieger, J. L., & Stellefson, M. L. (2017). The influence of eHealth literacy on perceived trust in online health communication channels and sources. *Journal of Health Communication*, *22*(1), 53-65.

[97] Pan, C.-H. (2006, June 19). *The effects of source cue and framing on consumers’ web-based health information processing*. Paper presented at the annual conference of the International Communication Association, Dresden, Germany.

[98] Park, S. Y., & Go, E. (2016). Health information seeking on the Internet: The role of involvement in searching for and assessing online health information. *Health Marketing Quarterly*, *33*(4), 327-341.

[99-100] Park, J., Chung, H., & Yoo, W. S. (2009). Is the Internet a primary source for consumer information search?: Group comparison for channel choices. *Journal of Retailing and Consumer Services*, *16*(2), 92-99.

[101] Peng, Z., & Logan, R. (2005). Content Quality, Usability, Affective Evaluation, and Overall Satisfaction of Online Health Information. *International Communication Association 2005 Annual Meeting*, New York, NY, p.1–31.

[102] Phua, J., & Tinkham, S. (2016). Authenticity in Obesity Public Service Announcements: Influence of Spokesperson Type, Viewer Weight, and Source Credibility on Diet, Exercise, Information Seeking, and Electronic Word-of-Mouth Intentions. *Journal of Health Communication*, *21*(3), 337–345.

[103] Purcarea, V. L., Gheorghe, I. R., & Petrescu, C. M. (2013). Credibility elements of eWOM messages in the context of health care services. A Romanian perspective. *Journal of Medicine and Life*, *6*(3), 254.

[104] Qiu, Y., Ren, W., Liu, Y., Yin, P., & Ren, J. (2019). Online health information in a rural residential population in Zhejiang Province, China: a cross-sectional study. *BMJ Open*, *9*(5), e026202.

[105] Rains, S. A. (2007). The anonymity effect: The influence of anonymity on perceptions of sources and information on health websites. *Journal of Applied Communication Research*, *35*(2), 197-214.

[106] Rains, S. A. (2008). Seeking health information in the information age: The role of Internet self-efficacy. *Western Journal of Communication*, *72*(1), 1-18.

[107] Rains, S. A., & Karmikel, C. D. (2009). Health information-seeking and perceptions of website credibility: Examining Web-use orientation, message characteristics, and structural features of websites. *Computers in Human Behavior*, *25*(2), 544-553.

[108] Ray, R., Sewell, A. A., Gilbert, K. L., & Roberts, J. D. (2017). Missed opportunity? Leveraging mobile technology to reduce racial health disparities. *Journal of Health Politics, Policy and Law*, *42*(5), 901-924.

[109] Record, R. A., Silberman, W. R., Santiago, J. E., & Ham, T. (2018). I sought it, I Reddit: Examining health information engagement behaviors among Reddit users. *Journal of Health Communication*, *23*(5), 470-476.

[110] Robertson-Lang, L., Major, S., & Hemming, H. (2011). An exploration of search patterns and credibility issues among older adults seeking online health information. *Canadian Journal on Aging/La Revue Canadienne du Vieillissement*, *30*(4), 631-645.

[111] Robins, D., Holmes, J., & Stansbury, M. (2010). Consumer health information on the Web: The relationship of visual design and perceptions of credibility. *Journal of the American Society for Information Science and Technology*, *61*(1), 13-29.

[112] Rutten, L. J. F., Blake, K., Hesse, B. W., & Ackerson, L. K. (2011). Isolated and skeptical: social engagement and trust in information sources among smokers. *Journal of Cancer Education*, *26*(3), 465-473.

[113] Sauls, M. E. (2018). *Perceived Credibility of Information on Internet Health Forums*. Clemson University. Retrieved February 19, 2022, from <https://www.proquest.com/docview/2056852831/abstract/703A311B27E541EEPQ/1>

[114] Sbaffi, L., & Zhao, C. (2020). Modeling the online health information seeking process: Information channel selection among university students. *Journal of the Association for Information Science and Technology*, *71*(2), 196-207.

[115] Schaefer, K. (2008). Perceived Credibility of Online Health Information: Application of a Health Consciousness Measure. *National Communication Association*, p.1-38.

[116] Schweiger, S., & Cress, U. (2019). How Confidence in Prior Attitudes, Social Tag Popularity, and Source Credibility Shape Confirmation Bias Toward Antidepressants and Psychotherapy in a Representative German Sample: Randomized Controlled Web-Based Study. *Journal of Medical Internet Research*, *21*(4), e11081.

[117] Seçkin, G. (2020). Expansion of Parson's sick role into cyberspace: Patient information consumerism and subjective health in a representative sample of US internet users. *Social Science & Medicine*, *247*, 112733.

[118] Selsky, C., Luta, G., Noone, A. M., Huerta, E. E., & Mandelblatt, J. S. (2013). Internet access and online cancer information seeking among Latino immigrants from safety net clinics. *Journal of Health Communication*, *18*(1), 58-70.

[119] Shakir, S. M. M., Wong, L. P., Abdullah, K. L., & Adam, P. (2019). Factors associated with online sexually transmissible infection information seeking among young people in Malaysia: an observational study. *Sexual Health*, *16*(2), 158-171.

[120] Shen, H., Xu, J., & Wang, Y. (2019). Applying situational theory of problem solving in cancer information seeking: A cross-sectional analysis of 2014 HINTS survey. *Journal of Health Communication*, *24*(2), 165-173.

[121] Sheng, X., & Simpson, P. M. (2015). Health care information seeking and seniors: determinants of Internet use. *Health Marketing Quarterly*, *32*(1), 96-112.

[122] Shim, M., & Jo, H. S. (2020). What quality factors matter in enhancing the perceived benefits of online health information sites? Application of the updated DeLone and McLean Information Systems Success Model. *International Journal of Medical Informatics*, *137*, 104093.

[123] Shon, J., Marshall, J., & Musen, M. A. (2000). The impact of displayed awards on the credibility and retention of web site information. *Proceedings of the AMIA Symposium* (p. 794). American Medical Informatics Association.

[124-125] Sillence, E., Blythe, J. M., Briggs, P., & Moss, M. (2019). A revised model of trust in internet-based health information and advice: cross-sectional questionnaire study. *Journal of Medical Internet Research*, *21*(11), e11125.

[126] Song, H., Omori, K., Kim, J., Tenzek, K. E., Hawkins, J. M., Lin, W. Y., ... & Jung, J. Y. (2016). Trusting social media as a source of health information: online surveys comparing the United States, Korea, and Hong Kong. *Journal of Medical Internet Research*, *18*(3), e4193.

[127] Song, S., Zhao, Y., Song, X., & Zhu, Q. (2019, June). The role of health literacy on credibility judgment of online health misinformation. *2019 IEEE International Conference on Healthcare Informatics (ICHI)* (pp. 1-3). IEEE.

[128] Song, X., Zhao, Y., Song, S., & Zhu, Q. (2019). The role of information cues on users' perceived credibility of online health rumors. *Proceedings of the Association for Information Science and Technology*, *56*(1), 760-761.

[129] Spence, P. R., Lachlan, K. A., Westerman, D., & Spates, S. A. (2013). Where the gates matter less: Ethnicity and perceived source credibility in social media health messages. *Howard Journal of Communications*, *24*(1), 1-16.

[130] Syn, S. Y., & Kim, S. U. (2013). The impact of source credibility on young adults' Health information activities on facebook: Preliminary findings. *Proceedings of the American Society for Information Science and Technology*, *50*(1), 1-4.

[131] Tao, D., Yuan, J., Shao, F., Li, D., Zhou, Q., & Qu, X. (2018). Factors affecting consumer acceptance of an online health information portal among young internet users. *CIN: Computers, Informatics, Nursing*, *36*(11), 530-539.

[132] Tedesco, J., & Holloway, R. (2005). Deceptive Health Promotion: Barriers to Health Literacy. *Studies in Communication Sciences*, *5*(2), 99-110.

[133] Thombre, A. (2004). *Analysis of message effectiveness of selected cancer Web sites*. The University of New Mexico. Retrieved February 19, 2022, from <https://www.proquest.com/docview/305164350/abstract/BDB700D81D6847A6PQ/1>

[134] Thomm, E., & Bromme, R. (2012). “It should at least seem scientific!” Textual features of “scientificness” and their impact on lay assessments of online information. *Science Education*, *96*(2), 187-211.

[135] Thompson, S., & Hickerson, C. (2007). Communicating Health Information in a Wiki-Environment: A Pilot Study Investigating the Health Wiki User and Health Wiki Sites. *National Communication Association*, p.1-26.

[136] Thon, F. M., & Jucks, R. (2017). Believing in expertise: How authors’ credentials and language use influence the credibility of online health information. *Health Communication*, *32*(7), 828-836.

[137] Van de Belt, T. H., Engelen, L. J., Berben, S. A., Teerenstra, S., Samsom, M., & Schoonhoven, L. (2013). Internet and social media for health-related information and communication in health care: preferences of the Dutch general population. *Journal of Medical Internet Research*, *15*(10), e2607.

[138] Van de Vord, R. (2007). Use of online health information for self and others: Predictors of credibility and verification by college students. *Annual Meeting of the International Communication Association, San Francisco, CA*.

[139] Walther, J. B., Jang, J. W., & Hanna Edwards, A. A. (2018). Evaluating health advice in a Web 2.0 environment: The impact of multiple user-generated factors on HIV advice perceptions. *Health Communication*, *33*(1), 57-67.

[140] Wang, Z., Walther, J. B., Pingree, S., & Hawkins, R. P. (2008). Health information, credibility, homophily, and influence via the Internet: Web sites versus discussion groups. *Health Communication*, *23*(4), 358-368.

[141] Williams, S. L., Ames, K., & Lawson, C. (2019). Preferences and trust in traditional and non-traditional sources of health information–a study of middle to older aged Australian adults. *Journal of Communication in Healthcare*, *12*(2), 134-142.

[142] Yang, F., & Li, C. (2016). The color of gender stereotyping: The congruity effect of topic, color, and gender on health messages’ persuasiveness in cyberspace. *Computers in Human Behavior*, *64*, 299-307.

[143] Ye, Y. (2010). A path analysis on correlates of consumer trust in online health information: evidence from the health information national trends survey. *Journal of Health Communication*, *15*(sup3), 200-215.

[144] Yun, E. K., & Park, H. A. (2010). Consumers’ disease information–seeking behaviour on the Internet in Korea. *Journal of Clinical Nursing*, *19*(19‐20), 2860-2868.

[145] Zimmermann, M., & Jucks, R. (2018). Investigating the Role of Communication for Information Seekers’ Trust-Related Evaluations of Health Videos on the Web: Content Analysis, Survey Data, and Experiment. *Interactive Journal of Medical Research*, *7*(2), e10282.

[146] Zimmermann, M., & Jucks, R. (2018). How experts’ use of medical technical jargon in different types of online health forums affects perceived information credibility: Randomized experiment with laypersons. *Journal of Medical Internet Research*, *20*(1), e8346.

[147] Zulman, D. M., Kirch, M., Zheng, K., & An, L. C. (2011). Trust in the internet as a health resource among older adults: analysis of data from a nationally representative survey. *Journal of Medical Internet Research*, *13*(1), e1552.
