# Supplementary material for: Consumers’ Evaluation of Web-Based Health Information Quality: Meta-analysis
Source: J Med Internet Res. 2022 Apr 28;24(4):e36463. doi: 10.2196/36463 (PMC9100526; doi:10.2196/36463)
Supplement: Multimedia Appendix 2 [file jmir_v24i4e36463_app2.docx]

**Multimedia Appendix II. Mean reliabilities**

| **Variable** | ***k*** | ***N*** | ***R_XX_*** |
| --- | --- | --- | --- |
| Quality of online health information | 87 | 33,252 | .85 |
| Attitude | 10 | 2,013 | .89 |
| Navigability | 9 | 2,639 | .81 |
| Aesthetics | 6 | 3,045 | .86 |
| Personal involvement | 6 | 2,130 | .85 |
| Intentions to use health information | 6 | 1,175 | .86 |
| Ease-of-understanding | 6 | 1,103 | .83 |
| Perceived usefulness | 5 | 4,190 | .89 |
| Source expertise | 5 | 3,864 | .88 |
| Health information use | 4 | 4,584 | .81 |
| Health information-seeking | 4 | 2,296 | .83 |
| Condition experience and beliefs | 3 | 6,321 | .70 |
| Health literacy | 3 | 2,591 | .89 |
| Source trustworthiness | 3 | 960 | .89 |
| Satisfaction with health information | 3 | 589 | .92 |
| Health knowledge | 2 | 772 | .86 |
| Perceived health benefits | 2 | 749 | .89 |
| Intentions to use health information systems | 1 | 255 | .88 |
| Internet experience | 1 | 151 | .93 |
| Content comprehensiveness | 1 | 139 | .84 |

*Note*. *k*=number of samples; *N*=total sample size; *R_XX_*=mean reliability.
