# Supplementary material for: Consumers’ Evaluation of Web-Based Health Information Quality: Meta-analysis
Source: J Med Internet Res. 2022 Apr 28;24(4):e36463. doi: 10.2196/36463 (PMC9100526; doi:10.2196/36463)
Supplement: Multimedia Appendix 3 [file jmir_v24i4e36463_app3.docx]

**Multimedia Appendix 3. Influence of moderators on the relationship between gender and web-based health IQ**

|  |  |  |  |  |  | **95% CI** | | **90% CV** | |  |  |  |  |
| --- | --- | --- | --- | --- | --- | --- | --- | --- | --- | --- | --- | --- | --- |
| **Moderators** | ***k*** | ***N*** | ***r*** | ***ρ*** | ***SD*** | **L** | **U** | **L** | **U** | ***Q_M_*** | ***Q_E_*** | ***I^2^*** | ***R^2^*** |
| **Technology Context** | |  |  |  |  |  |  |  |  |  |  |  |  |
| Social media | 4 | 2,092 | -.09 | -.10 | .13 | -.23 | .02 | -.31 | .11 | 4.55* | 157.89** | 81.23% | 30.53% |
| Non-social media | 21 | 18,009 | .06 | .06 | .12 | -.01 | .14 | -.14 | .26 |  |  |  |  |
| **Individualism vs. Collectivism** | | | |  |  |  |  |  |  |  |  |  |  |
| Individualism | 18 | 17,745 | .06 | .06 | .11 | -.02 | .14 | -.12 | .24 | 10.50** | 155.56** | 83.18% | 23.33% |
| Collectivism | 4 | 1,242 | -.10 | -.11 | .11 | -.23 | .00 | -.30 | .08 |  |  |  |  |
| **Power Distance** |  |  |  |  |  |  |  |  |  |  |  |  |  |
| High | 5 | 1,901 | -.02 | -.02 | .16 | -.18 | .14 | -.29 | .25 | 3.85* | 183.26** | 85.45% | 7.29% |
| Low | 17 | 17,086 | .05 | .06 | .11 | -.03 | .14 | -.12 | .24 |  |  |  |  |
| **Uncertainty Avoidance** | | |  |  |  |  |  |  |  |  |  |  |  |
| High | 10 | 13,180 | .08 | .08 | .08 | .03 | .14 | -.05 | .21 | 6.78** | 138.71** | 79.32% | 32.90% |
| Low | 12 | 5,807 | -.02 | -.03 | .15 | -.12 | .06 | -.27 | .21 |  |  |  |  |
| **Orientation** |  |  |  |  |  |  |  |  |  |  |  |  |  |
| Long-term | 15 | 14,013 | .07 | .07 | .14 | -.03 | .17 | -.16 | .30 | .57 | 169.15** | 83.10% | 15.40% |
| Short-term | 7 | 4,974 | -.01 | -.01 | .11 | -.10 | .08 | -.19 | .17 |  |  |  |  |
| **Indulgence vs. Restraint** | | |  |  |  |  |  |  |  |  |  |  |  |
| Indulgence | 10 | 6,213 | .00 | .00 | .12 | -.08 | .08 | -.20 | .20 | .00 | 172.34** | 82.96% | 13.57% |
| Restraint | 12 | 12,774 | .07 | .07 | .14 | -.03 | .18 | -.16 | .30 |  |  |  |  |
| **Focal Variable** | | | | | | | | | | | | | |
| Credibility | 9 | 2,867 | .02 | .02 | .16 | -.08 | .13 | -.24 | .28 | .07 | 183.03** | 87.33% | 1.94% |
| Trust | 9 | 14,572 | .05 | .06 | .12 | -.05 | .16 | -.14 | .26 |  |  |  |  |
| **Sample Clinical Status** | | |  |  |  |  |  |  |  |  |  |  |  |
| Patients | 4 | 9,478 | .08 | .09 | .09 | .05 | .13 | -.06 | .24 | 7.18** | 171.21** | 78.65% | 23.57% |
| Non-patients | 21 | 10,623 | .00 | .00 | .12 | -.06 | .06 | -.19 | .19 |  |  |  |  |
| **Sample Type** |  |  |  |  |  |  |  |  |  |  |  |  |  |
| Students | 7 | 1,978 | .01 | .01 | .07 | -.04 | .05 | -.10 | .12 | .00 | 213.72** | 86.23% | 1.34% |
| Non-students | 18 | 18,123 | .04 | .05 | .14 | -.05 | .15 | -.19 | .29 |  |  |  |  |
| **Study Method** |  |  |  |  |  |  |  |  |  |  |  |  |  |
| Survey | 17 | 17,929 | .05 | .06 | .12 | -.03 | .14 | -.14 | .26 | 5.39* | 184.65** | 84.07% | 16.54% |
| Experiment | 8 | 2,172 | -.06 | -.06 | .12 | -.15 | .02 | -.25 | .13 |  |  |  |  |
| **Stimulus Type** |  |  |  |  |  |  |  |  |  |  |  |  |  |
| General | 13 | 16,088 | .05 | .06 | .12 | -.04 | .15 | -.14 | .26 | 2.13 | 200.78** | 84.88% | 8.11% |
| Specific | 12 | 4,013 | -.01 | -.01 | .13 | -.09 | .07 | -.22 | .20 |  |  |  |  |
| **Publication Outlet** |  |  |  |  |  |  |  |  |  |  |  |  |  |
| Journal | 17 | 18,204 | .05 | .06 | .12 | -.03 | .14 | -.13 | .25 | 6.41* | 178.26** | 83.62% | 19.88% |
| Non-journal | 8 | 1,897 | -.08 | -.08 | .12 | -.16 | -.00 | -.27 | .11 |  |  |  |  |
| **Publication Year** |  |  |  |  |  |  |  |  |  |  |  |  |  |
| Prior to 2014 | 9 | 12,145 | .07 | .08 | .09 | .03 | .12 | -.06 | .22 | 5.26* | 175.96** | 81.16% | 21.08% |
| 2014 and after | 16 | 7,956 | -.01 | -.01 | .13 | -.08 | .07 | -.23 | .21 |  |  |  |  |

*Note*. *k*=number of samples; *N*=total sample size; *r*=weighted mean correlation; *ρ*=weighted mean correlation corrected for measurement unreliability; SD=standard deviation of *ρ*; 95% CI=lower and upper limits of 95% confidence interval; 90% CV=lower and upper limits of 90% credibility interval; *Q_M_*=moderator test; *Q_E_*=amount of observed heterogeneity unexplained by the moderator; *I^2^*=percentage of variation across studies that is due to heterogeneity; *R^2^*=percent of variation explained by random-effects regression model.

***p*<.01, **p*<.05.
