# Supplementary material for: Consumers’ Evaluation of Web-Based Health Information Quality: Meta-analysis
Source: J Med Internet Res. 2022 Apr 28;24(4):e36463. doi: 10.2196/36463 (PMC9100526; doi:10.2196/36463)
Supplement: Multimedia Appendix 4 [file jmir_v24i4e36463_app4.docx]

**Multimedia Appendix 4. Influence of moderators on the relationship between age and web-based health IQ**

|  |  |  |  |  |  | **95% CI** | | **90% CV** | |  |  |  |  |
| --- | --- | --- | --- | --- | --- | --- | --- | --- | --- | --- | --- | --- | --- |
| **Moderators** | ***k*** | ***N*** | ***r*** | ***ρ*** | ***SD*** | **L** | **U** | **L** | **U** | ***Q_M_*** | ***Q_E_*** | ***I^2^*** | ***R^2^*** |
| **Technology Context** | |  |  |  |  |  |  |  |  |  |  |  |  |
| Social media | 3 | 1,397 | .02 | .02 | .09 | -.05 | .09 | -.12 | .16 | .06 | 832.86** | 97.17% | .24% |
| Non-social media | 17 | 22,066 | .04 | .04 | .14 | -.13 | .22 | -.19 | .27 |  |  |  |  |
| **Individualism vs. Collectivism** | | | |  |  |  |  |  |  |  |  |  |  |
| Individualism | 12 | 19,834 | .05 | .05 | .13 | -.14 | .24 | -.16 | .26 | .32 | 794.34** | 97.37% | .26% |
| Collectivism | 5 | 2,542 | .02 | .03 | .15 | -.19 | .24 | -.22 | .28 |  |  |  |  |
| **Power Distance** |  |  |  |  |  |  |  |  |  |  |  |  |  |
| High | 6 | 3,201 | .01 | .01 | .15 | -.16 | .18 | -.23 | .25 | .07 | 788.06** | 97.33% | 1.07% |
| Low | 11 | 19,175 | .05 | .05 | .13 | -.14 | .25 | -.16 | .26 |  |  |  |  |
| **Uncertainty Avoidance** | | |  |  |  |  |  |  |  |  |  |  |  |
| High | 8 | 12,795 | -.07 | -.07 | .08 | -.11 | -.03 | -.21 | .07 | 7.37** | 286.63** | 90.93% | 65.40% |
| Low | 9 | 9,581 | .19 | .21 | .13 | .02 | .39 | -.01 | .43 |  |  |  |  |
| **Orientation** |  |  |  |  |  |  |  |  |  |  |  |  |  |
| Long-term | 13 | 14,411 | -.04 | -.05 | .12 | -.16 | .06 | -.24 | .14 | 2.93 | 359.51** | 92.67% | 56.05% |
| Short-term | 4 | 7,965 | .20 | .22 | .17 | -.00 | .44 | -.06 | .50 |  |  |  |  |
| **Indulgence vs. Restraint** | | |  |  |  |  |  |  |  |  |  |  |  |
| Indulgence | 6 | 8,974 | .17 | .19 | .15 | -.02 | .40 | -.06 | .44 | 3.86* | 421.47** | 93.77% | 48.10% |
| Restraint | 11 | 13,402 | -.04 | -.05 | .13 | -.17 | .07 | -.26 | .16 |  |  |  |  |
| **Focal Variable** | | | | | | | | | | | | | |
| Quality | 3 | 737 | .04 | .04 | .05 | -.03 | .11 | -.04 | .12 | .94 | 378.32** | 92.50% | 55.59% |
| Credibility | 8 | 7,801 | .20 | .22 | .15 | -.01 | .45 | -.03 | .47 |  |  |  |  |
| Trust | 8 | 14,290 | -.05 | -.05 | .14 | -.16 | .06 | -.29 | .19 |  |  |  |  |
| **Sample Type** |  |  |  |  |  |  |  |  |  |  |  |  |  |
| Students | 3 | 594 | -.03 | -.03 | .10 | -.13 | .06 | -.19 | .13 | .03 | 829.67** | 97.23% | .63% |
| Non-students | 17 | 22,869 | .04 | .04 | .14 | -.12 | .21 | -.19 | .27 |  |  |  |  |
| **Study Method** |  |  |  |  |  |  |  |  |  |  |  |  |  |
| Survey | 13 | 21,681 | .04 | .04 | .15 | -.13 | .22 | -.20 | .30 | .01 | 825.19** | 97.17% | 1.18% |
| Experiment | 7 | 1,782 | -.01 | -.01 | .11 | -.11 | .09 | -.19 | .17 |  |  |  |  |
| **Stimulus Type** |  |  |  |  |  |  |  |  |  |  |  |  |  |
| General | 11 | 20,648 | .05 | .05 | .16 | -.13 | .24 | -.21 | .31 | .05 | 813.63** | 97.08% | 2.60% |
| Specific | 9 | 2,815 | -.02 | -.02 | .10 | -.10 | .05 | -.19 | .15 |  |  |  |  |
| **Publication Outlet** |  |  |  |  |  |  |  |  |  |  |  |  |  |
| Journal | 12 | 21,288 | .04 | .05 | .16 | -.13 | .23 | -.21 | .31 | .02 | 821.50** | 97.14% | 1.64% |
| Non-journal | 8 | 2,175 | -.01 | -.01 | .10 | -.09 | .07 | -.17 | .15 |  |  |  |  |
| **Publication Year** |  |  |  |  |  |  |  |  |  |  |  |  |  |
| Prior to 2014 | 7 | 16,892 | .06 | .07 | .14 | -.16 | .29 | -.16 | .30 | .15 | 788.05** | 96.76% | 5.74% |
| 2014 and after | 13 | 6,571 | -.02 | -.02 | .13 | -.11 | .07 | -.24 | .20 |  |  |  |  |

*Note*. *k*=number of samples; *N*=total sample size; *r*=weighted mean correlation; *ρ*=weighted mean correlation corrected for measurement unreliability; SD=standard deviation of *ρ*; 95% CI=lower and upper limits of 95% confidence interval; 90% CV=lower and upper limits of 90% credibility interval; *Q_M_*=moderator test; *Q_E_*=amount of observed heterogeneity unexplained by the moderator; *I^2^*=percentage of variation across studies that is due to heterogeneity; *R^2^*=percent of variation explained by random-effects regression model.

***p*<.01, **p*<.05.
