# Supplementary material for: Consumers’ Evaluation of Web-Based Health Information Quality: Meta-analysis
Source: J Med Internet Res. 2022 Apr 28;24(4):e36463. doi: 10.2196/36463 (PMC9100526; doi:10.2196/36463)
Supplement: Multimedia Appendix 5 [file jmir_v24i4e36463_app5.docx]

**Multimedia Appendix 5. Influence of moderators on the relationship between source-related factors and web-based health IQ**

|  |  |  |  |  |  | **95% CI** | | **90% CV** | |  |  |  |  |
| --- | --- | --- | --- | --- | --- | --- | --- | --- | --- | --- | --- | --- | --- |
| **Moderators** | ***k*** | ***N*** | ***r*** | ***ρ*** | ***SD*** | **L** | **U** | **L** | **U** | ***Q_M_*** | ***Q_E_*** | ***I^2^*** | ***R^2^*** |
| **Technology Context** | |  |  |  |  |  |  |  |  |  |  |  |  |
| Social media | 10 | 2,011 | .28 | .32 | .23 | .11 | .54 | -.06 | .70 | 3.33 | 1780.15** | 98.08% | 2.07% |
| Non-social media | 20 | 8,131 | .18 | .21 | .24 | -.04 | .47 | -.18 | .60 |  |  |  |  |
| **Individualism vs. Collectivism** | | | |  |  |  |  |  |  |  |  |  |  |
| Individualism | 21 | 7,645 | .19 | .22 | .24 | .01 | .43 | -.18 | .62 | 3.20 | 1015.16** | 96.57% | 38.54% |
| Collectivism | 6 | 1,460 | .40 | .44 | .27 | .24 | .64 | -.00 | .88 |  |  |  |  |
| **Power Distance** |  |  |  |  |  |  |  |  |  |  |  |  |  |
| High | 6 | 1,460 | .40 | .44 | .27 | .24 | .64 | -.00 | .88 | 3.20 | 1015.16** | 96.57% | 38.54% |
| Low | 21 | 7,645 | .19 | .22 | .24 | .01 | .43 | -.18 | .62 |  |  |  |  |
| **Uncertainty Avoidance** | | |  |  |  |  |  |  |  |  |  |  |  |
| High | 14 | 3,022 | .33 | .37 | .26 | .21 | .53 | -.05 | .79 | 6.25* | 864.19** | 96.15% | 47.93% |
| Low | 13 | 6,083 | .17 | .20 | .21 | .04 | .35 | -.15 | .55 |  |  |  |  |
| **Orientation** |  |  |  |  |  |  |  |  |  |  |  |  |  |
| Long-term | 19 | 4,457 | .28 | .32 | .25 | .16 | .48 | -.10 | .74 | 7.21** | 1051.48** | 96.79% | 36.28% |
| Short-term | 8 | 4,648 | .16 | .19 | .12 | .09 | .29 | -.00 | .38 |  |  |  |  |
| **Indulgence vs. Restraint** | |  |  |  |  |  |  |  |  |  |  |  |  |
| Indulgence | 9 | 5,223 | .14 | .17 | .11 | .06 | .28 | -.01 | .35 | 11.47** | 859.44** | 96.10% | 48.22% |
| Restraint | 18 | 3,882 | .32 | .37 | .24 | .22 | .51 | -.03 | .77 |  |  |  |  |
| **Focal Variable** | | | | | | | | | | | | | |
| Quality | 8 | 1,899 | .28 | .30 | .32 | .08 | .53 | -.23 | .83 | .09 | 1297.10** | 96.69% | 29.10% |
| Credibility | 18 | 4,859 | .17 | .20 | .26 | .03 | .37 | -.27 | .63 |  |  |  |  |
| Trust | 4 | 3,384 | .21 | .25 | .13 | .18 | .31 | .03 | .47 |  |  |  |  |
| **Sample Type** |  |  |  |  |  |  |  |  |  |  |  |  |  |
| Students | 13 | 2,959 | .20 | .22 | .19 | .09 | .35 | -.10 | .54 | .00 | 1665.34** | 98.00% | 8.49% |
| Non-students | 17 | 7,183 | .21 | .24 | .30 | -.05 | .53 | -.26 | .74 |  |  |  |  |
| **Study Method** |  |  |  |  |  |  |  |  |  |  |  |  |  |
| Survey | 7 | 4,884 | .21 | .25 | .28 | -.08 | .58 | -.22 | .72 | .57 | 1758.45** | 97.93% | 3.28% |
| Experiment | 23 | 5,258 | .20 | .22 | .25 | .06 | .39 | -.19 | .63 |  |  |  |  |
| **Stimulus Type** |  |  |  |  |  |  |  |  |  |  |  |  |  |
| General | 4 | 1,321 | .29 | .35 | .31 | .05 | .65 | -.16 | .86 | 1.31 | 1744.18** | 98.03% | 4.08% |
| Specific | 26 | 8,821 | .19 | .22 | .25 | -.02 | .45 | -.18 | .62 |  |  |  |  |
| **Publication Outlet** |  |  |  |  |  |  |  |  |  |  |  |  |  |
| Journal | 20 | 7,528 | .21 | .24 | .28 | -.03 | .51 | -.22 | .70 | .18 | 1685.14** | 98.02% | 7.38% |
| Non-journal | 10 | 2,614 | .18 | .21 | .22 | .05 | .37 | -.15 | .57 |  |  |  |  |
| **Publication Year** |  |  |  |  |  |  |  |  |  |  |  |  |  |
| Prior to 2014 | 13 | 5,463 | .23 | .26 | .27 | -.08 | .61 | -.19 | .71 | .00 | 1738.51** | 97.98% | 4.40% |
| 2014 and after | 17 | 4,679 | .17 | .20 | .26 | .02 | .37 | -.22 | .62 |  |  |  |  |

*Note*. *k*=number of samples; *N*=total sample size; *r*=weighted mean correlation; *ρ*=weighted mean correlation corrected for measurement unreliability; SD=standard deviation of *ρ*; 95% CI=lower and upper limits of 95% confidence interval; 90% CV=lower and upper limits of 90% credibility interval; *Q_M_*=moderator test; *Q_E_*=amount of observed heterogeneity unexplained by the moderator; *I^2^*=percentage of variation across studies that is due to heterogeneity; *R^2^*=percent of variation explained by random-effects regression model.

***p*<.01, **p*<.05.
