# Supplementary material for: Consumers’ Evaluation of Web-Based Health Information Quality: Meta-analysis
Source: J Med Internet Res. 2022 Apr 28;24(4):e36463. doi: 10.2196/36463 (PMC9100526; doi:10.2196/36463)
Supplement: Multimedia Appendix 6 [file jmir_v24i4e36463_app6.docx]

**Multimedia Appendix 6. Influence of moderators on the relationship between content-related factors and web-based health IQ**

|  |  |  |  |  |  | **95% CI** | | **90% CV** | |  |  |  |  |
| --- | --- | --- | --- | --- | --- | --- | --- | --- | --- | --- | --- | --- | --- |
| **Moderators** | ***k*** | ***N*** | ***r*** | ***ρ*** | ***SD*** | **L** | **U** | **L** | **U** | ***Q_M_*** | ***Q_E_*** | ***I^2^*** | ***R^2^*** |
| **Technology Context** | |  |  |  |  |  |  |  |  |  |  |  |  |
| Social media | 10 | 2,493 | .20 | .24 | .29 | -.02 | .49 | -.24 | .72 | .72 | 1704.37** | 98.25% | 3.20% |
| Non-social media | 17 | 4,550 | .35 | .41 | .31 | .26 | .56 | -.10 | .92 |  |  |  |  |
| **Individualism vs. Collectivism** | | | |  |  |  |  |  |  |  |  |  |  |
| Individualism | 20 | 5,491 | .31 | .37 | .31 | .22 | .52 | -.15 | .89 | .03 | 1490.71** | 98.23% | .23% |
| Collectivism | 4 | 468 | .41 | .46 | .32 | .22 | .70 | -.06 | .98 |  |  |  |  |
| **Power Distance** |  |  |  |  |  |  |  |  |  |  |  |  |  |
| High | 4 | 468 | .41 | .46 | .32 | .22 | .70 | -.06 | .98 | .03 | 1490.71** | 98.23% | .23% |
| Low | 20 | 5,491 | .31 | .37 | .31 | .22 | .52 | -.15 | .89 |  |  |  |  |
| **Uncertainty Avoidance** | | |  |  |  |  |  |  |  |  |  |  |  |
| High | 11 | 2,177 | .38 | .45 | .27 | .27 | .63 | -.00 | .90 | .13 | 1491.85** | 98.19% | .16% |
| Low | 13 | 3,782 | .28 | .33 | .34 | .14 | .52 | -.24 | .90 |  |  |  |  |
| **Orientation** |  |  |  |  |  |  |  |  |  |  |  |  |  |
| Long-term | 15 | 3,551 | .40 | .48 | .28 | .34 | .62 | .01 | .95 | .52 | 1486.75** | 98.16% | .50% |
| Short-term | 9 | 2,408 | .19 | .22 | .36 | -.05 | .50 | -.37 | .81 |  |  |  |  |
| **Indulgence vs. Restraint** | | |  |  |  |  |  |  |  |  |  |  |  |
| Indulgence | 11 | 3,582 | .29 | .34 | .36 | .14 | .54 | -.25 | .93 | .13 | 1486.86** | 98.26% | .00% |
| Restraint | 12 | 2,307 | .36 | .43 | .28 | .25 | .61 | -.04 | .90 |  |  |  |  |
| **Focal Variable** | | | | | | | | | | | | | |
| Quality | 6 | 1,632 | .27 | .31 | .38 | .03 | .58 | -.31 | .93 | 2.19 | 1702.70** | 98.13% | 3.30% |
| Credibility | 16 | 3,943 | .25 | .29 | .30 | .10 | .48 | -.20 | .78 |  |  |  |  |
| Trust | 5 | 1,468 | .46 | .57 | .20 | .42 | .72 | .25 | .89 |  |  |  |  |
| **Sample Type** |  |  |  |  |  |  |  |  |  |  |  |  |  |
| Students | 13 | 2,947 | .21 | .24 | .24 | .01 | .47 | -.16 | .64 | 5.63* | 1602.67** | 98.15% | 9.07% |
| Non-students | 14 | 4,096 | .36 | .43 | .31 | .27 | .58 | -.08 | .93 |  |  |  |  |
| **Study Method** |  |  |  |  |  |  |  |  |  |  |  |  |  |
| Survey | 10 | 3,271 | .43 | .51 | .26 | .36 | .66 | .08 | .94 | 9.55** | 1594.84** | 98.11% | 9.52% |
| Experiment | 17 | 3,772 | .18 | .21 | .25 | .00 | .41 | -.20 | .62 |  |  |  |  |
| **Stimulus Type** |  |  |  |  |  |  |  |  |  |  |  |  |  |
| General | 5 | 822 | .64 | .73 | .21 | .64 | .81 | .39 | 1.00 | 9.53** | 1375.35** | 97.79% | 22.19% |
| Specific | 22 | 6,221 | .25 | .30 | .27 | .14 | .46 | -.15 | .75 |  |  |  |  |
| **Publication Outlet** |  |  |  |  |  |  |  |  |  |  |  |  |  |
| Journal | 19 | 5,426 | .32 | .38 | .31 | .23 | .52 | -.12 | .88 | .73 | 1751.49** | 98.24% | .48% |
| Non-journal | 8 | 1,617 | .23 | .26 | .30 | -.02 | .53 | -.22 | .75 |  |  |  |  |
| **Publication Year** |  |  |  |  |  |  |  |  |  |  |  |  |  |
| Prior to 2014 | 8 | 1,684 | .29 | .33 | .35 | .08 | .57 | -.24 | .90 | .01 | 1708.18** | 98.21% | 2.98% |
| 2014 and after | 19 | 5,359 | .30 | .36 | .29 | .20 | .51 | -.12 | .84 |  |  |  |  |

*Note*. *k*=number of samples; *N*=total sample size; *r*=weighted mean correlation; *ρ*=weighted mean correlation corrected for measurement unreliability; SD=standard deviation of *ρ*; 95% CI=lower and upper limits of 95% confidence interval; 90% CV=lower and upper limits of 90% credibility interval; *Q_M_*=moderator test; *Q_E_*=amount of observed heterogeneity unexplained by the moderator; *I^2^*=percentage of variation across studies that is due to heterogeneity; *R^2^*=percent of variation explained by random-effects regression model.

***p*<.01, **p*<.05.
