# Supplementary material for: Consumers’ Evaluation of Web-Based Health Information Quality: Meta-analysis
Source: J Med Internet Res. 2022 Apr 28;24(4):e36463. doi: 10.2196/36463 (PMC9100526; doi:10.2196/36463)
Supplement: Multimedia Appendix 7 [file jmir_v24i4e36463_app7.docx]

**Multimedia Appendix 7. Influence of moderators on the relationship between design-related factors and web-based health IQ**

|  |  |  |  |  |  | **95% CI** | | **90% CV** | |  |  |  |  |
| --- | --- | --- | --- | --- | --- | --- | --- | --- | --- | --- | --- | --- | --- |
| **Moderators** | ***k*** | ***N*** | ***r*** | ***ρ*** | ***SD*** | **L** | **U** | **L** | **U** | ***Q_M_*** | ***Q_E_*** | ***I^2^*** | ***R^2^*** |
| **Individualism vs. Collectivism** | | | |  |  |  |  |  |  |  |  |  |  |
| Individualism | 9 | 2,127 | .33 | .39 | .25 | .20 | .59 | -.01 | .79 | .24 | 615.44** | 96.92% | 10.46% |
| Collectivism | 6 | 1,917 | .48 | .57 | .34 | .41 | .72 | .02 | 1.00 |  |  |  |  |
| **Power Distance** |  |  |  |  |  |  |  |  |  |  |  |  |  |
| High | 6 | 1,917 | .48 | .57 | .34 | .41 | .72 | .02 | 1.00 | .24 | 615.44** | 96.92% | 10.46% |
| Low | 9 | 2,127 | .33 | .39 | .25 | .20 | .59 | -.01 | .79 |  |  |  |  |
| **Uncertainty Avoidance** | | |  |  |  |  |  |  |  |  |  |  |  |
| High | 4 | 969 | .52 | .61 | .25 | .44 | .79 | .20 | 1.00 | .25 | 611.19** | 96.47% | 11.10% |
| Low | 11 | 3,075 | .37 | .43 | .29 | .27 | .59 | -.05 | .91 |  |  |  |  |
| **Orientation** |  |  |  |  |  |  |  |  |  |  |  |  |  |
| Long-term | 9 | 2,556 | .44 | .52 | .28 | .38 | .67 | .06 | .98 | .02 | 667.65** | 97.20% | 2.68% |
| Short-term | 6 | 1,488 | .33 | .39 | .30 | .15 | .64 | -.10 | .88 |  |  |  |  |
| **Indulgence vs. Restraint** | | |  |  |  |  |  |  |  |  |  |  |  |
| Indulgence | 7 | 1,862 | .35 | .41 | .27 | .20 | .62 | -.03 | .85 | .04 | 646.43** | 97.08% | 5.84% |
| Restraint | 8 | 2,182 | .45 | .53 | .30 | .38 | .68 | .04 | 1.00 |  |  |  |  |
| **Focal Variable** | | | | | | | | | | | | | |
| Quality | 10 | 4,815 | .48 | .58 | .24 | .44 | .72 | .18 | .98 | 9.34** | 795.37** | 97.11% | 10.22% |
| Credibility | 9 | 1,704 | .27 | .33 | .31 | .11 | .56 | -.18 | .84 |  |  |  |  |
| **Sample Type** |  |  |  |  |  |  |  |  |  |  |  |  |  |
| Students | 4 | 841 | .42 | .50 | .24 | .29 | .70 | .11 | .89 | .04 | 885.46** | 97.27% | .03% |
| Non-students | 16 | 5,851 | .43 | .52 | .32 | .37 | .67 | -.01 | 1.00 |  |  |  |  |
| **Study Method** |  |  |  |  |  |  |  |  |  |  |  |  |  |
| Survey | 14 | 5,795 | .46 | .56 | .30 | .42 | .69 | .06 | 1.00 | 2.46 | 830.80** | 97.24% | 6.34% |
| Experiment | 6 | 897 | .22 | .26 | .29 | -.03 | .56 | -.22 | .74 |  |  |  |  |
| **Stimulus Type** |  |  |  |  |  |  |  |  |  |  |  |  |  |
| General | 9 | 3,963 | .49 | .60 | .34 | .46 | .74 | .05 | 1.00 | .49 | 881.95** | 96.95% | .43% |
| Specific | 11 | 2,729 | .33 | .39 | .28 | .20 | .58 | -.08 | .86 |  |  |  |  |
| **Publication Outlet** |  |  |  |  |  |  |  |  |  |  |  |  |  |
| Journal | 13 | 4,751 | .40 | .50 | .29 | .33 | .68 | .03 | .97 | 2.85 | 882.20** | 97.11% | .40% |
| Non-journal | 7 | 1,941 | .48 | .54 | .32 | .40 | .69 | .01 | 1.00 |  |  |  |  |
| **Publication Year** |  |  |  |  |  |  |  |  |  |  |  |  |  |
| Prior to 2014 | 12 | 4,380 | .42 | .52 | .31 | .34 | .69 | .03 | 1.00 | .21 | 856.53** | 96.82% | 3.37% |
| 2014 and after | 8 | 2,312 | .44 | .52 | .30 | .37 | .67 | .02 | 1.00 |  |  |  |  |

*Note*. *k*=number of samples; *N*=total sample size; *r*=weighted mean correlation; *ρ*=weighted mean correlation corrected for measurement unreliability; SD=standard deviation of *ρ*; 95% CI=lower and upper limits of 95% confidence interval; 90% CV=lower and upper limits of 90% credibility interval; *Q_M_*=moderator test; *Q_E_*=amount of observed heterogeneity unexplained by the moderator; *I^2^*=percentage of variation across studies that is due to heterogeneity; *R^2^*=percent of variation explained by random-effects regression model.

***p*<.01, **p*<.05.
