# Supplementary material for: Consumers’ Evaluation of Web-Based Health Information Quality: Meta-analysis
Source: J Med Internet Res. 2022 Apr 28;24(4):e36463. doi: 10.2196/36463 (PMC9100526; doi:10.2196/36463)
Supplement: Multimedia Appendix 8 [file jmir_v24i4e36463_app8.docx]

**Multimedia Appendix 8. Influence of moderators on the relationship between web-based health IQ and cognitive appraisals**

|  |  |  |  |  |  | **95% CI** | | **90% CV** | |  |  |  |  |
| --- | --- | --- | --- | --- | --- | --- | --- | --- | --- | --- | --- | --- | --- |
| **Moderators** | ***k*** | ***N*** | ***r*** | ***ρ*** | ***SD*** | **L** | **U** | **L** | **U** | ***Q_M_*** | ***Q_E_*** | ***I^2^*** | ***R^2^*** |
| **Individualism vs. Collectivism** | | | |  |  |  |  |  |  |  |  |  |  |
| Individualism | 23 | 15,533 | .27 | .31 | .28 | .22 | .39 | -.15 | .77 | 1.88 | 9462.22** | 99.22% | 15.90% |
| Collectivism | 11 | 4,008 | .34 | .39 | .19 | .24 | .54 | .07 | .71 |  |  |  |  |
| **Power Distance** |  |  |  |  |  |  |  |  |  |  |  |  |  |
| High | 11 | 4,008 | .34 | .39 | .19 | .24 | .54 | .07 | .71 | 1.88 | 9462.22** | 99.22% | 15.90% |
| Low | 23 | 15,533 | .27 | .31 | .28 | .22 | .39 | -.15 | .77 |  |  |  |  |
| **Uncertainty Avoidance** | | |  |  |  |  |  |  |  |  |  |  |  |
| High | 17 | 7,284 | .28 | .31 | .25 | .23 | .40 | -.10 | .72 | 1.23 | 11230.41** | 95.56% | .13% |
| Low | 17 | 12,257 | .29 | .33 | .26 | .23 | .44 | -.10 | .76 |  |  |  |  |
| **Orientation** |  |  |  |  |  |  |  |  |  |  |  |  |  |
| Long-term | 23 | 7,935 | .35 | .40 | .22 | .34 | .47 | .04 | .76 | 3.88* | 11217.72** | 95.54% | .24% |
| Short-term | 11 | 11,606 | .24 | .27 | .31 | .16 | .39 | -.24 | .78 |  |  |  |  |
| **Indulgence vs. Restraint** | | |  |  |  |  |  |  |  |  |  |  |  |
| Indulgence | 13 | 11,190 | .27 | .31 | .29 | .20 | .42 | -.17 | .79 | .37 | 10251.03** | 95.06% | .15% |
| Restraint | 20 | 6,945 | .33 | .38 | .23 | .31 | .45 | .00 | .76 |  |  |  |  |
| **Focal Variable** | | | | | | | | | | | | | |
| Quality | 11 | 4,510 | .39 | .45 | .23 | .31 | .59 | .07 | .83 | 3.59 | 6482.04** | 98.77% | 49.41% |
| Credibility | 17 | 9,301 | .23 | .26 | .28 | .17 | .36 | -.20 | .72 |  |  |  |  |
| Trust | 9 | 9,205 | .31 | .36 | .20 | .23 | .50 | .03 | .69 |  |  |  |  |
| **Sample Type** |  |  |  |  |  |  |  |  |  |  |  |  |  |
| Students | 8 | 1,693 | .23 | .26 | .24 | .07 | .45 | -.13 | .65 | 9.20** | 11842.91** | 99.33% | 7.33% |
| Non-students | 29 | 21,323 | .30 | .35 | .24 | .27 | .42 | -.04 | .74 |  |  |  |  |
| **Study Method** |  |  |  |  |  |  |  |  |  |  |  |  |  |
| Survey | 27 | 21,314 | .29 | .34 | .24 | .25 | .42 | -.05 | .73 | .49 | 12687.30** | 96.31% | .70% |
| Experiment | 10 | 1,702 | .36 | .41 | .27 | .35 | .46 | -.03 | .85 |  |  |  |  |
| **Stimulus Type** |  |  |  |  |  |  |  |  |  |  |  |  |  |
| General | 18 | 15,477 | .24 | .27 | .24 | .17 | .37 | -.15 | .69 | 4.74* | 12770.23** | 96.34% | .05% |
| Specific | 19 | 7,539 | .42 | .48 | .23 | .39 | .58 | .10 | .86 |  |  |  |  |
| **Publication Outlet** |  |  |  |  |  |  |  |  |  |  |  |  |  |
| Journal | 30 | 21,454 | .29 | .33 | .25 | .26 | .41 | -.08 | .74 | .41 | 12158.84** | 99.35% | 4.85% |
| Non-journal | 7 | 1,562 | .40 | .45 | .22 | .29 | .60 | .08 | .82 |  |  |  |  |
| **Publication Year** |  |  |  |  |  |  |  |  |  |  |  |  |  |
| Prior to 2014 | 21 | 14,357 | .31 | .37 | .26 | .29 | .46 | -.10 | .80 | 6.42* | 8425.66** | 99.05% | 34.15% |
| 2014 and after | 16 | 8,659 | .28 | .32 | .21 | .20 | .44 | -.03 | .67 |  |  |  |  |

*Note*. *k*=number of samples; *N*=total sample size; *r*=weighted mean correlation; *ρ*=weighted mean correlation corrected for measurement unreliability; SD=standard deviation of *ρ*; 95% CI=lower and upper limits of 95% confidence interval; 90% CV=lower and upper limits of 90% credibility interval; *Q_M_*=moderator test; *Q_E_*=amount of observed heterogeneity unexplained by the moderator; *I^2^*=percentage of variation across studies that is due to heterogeneity; *R^2^*=percent of variation explained by random-effects regression model.

***p*<.01, **p*<.05.
