# Supplementary material for: Consumers’ Evaluation of Web-Based Health Information Quality: Meta-analysis
Source: J Med Internet Res. 2022 Apr 28;24(4):e36463. doi: 10.2196/36463 (PMC9100526; doi:10.2196/36463)
Supplement: Multimedia Appendix 9 [file jmir_v24i4e36463_app9.docx]

**Multimedia Appendix 9. Influence of moderators on the relationship between web-based health IQ and behavioral intentions**

|  |  |  |  |  |  | **95% CI** | | **90% CV** | |  |  |  |  |
| --- | --- | --- | --- | --- | --- | --- | --- | --- | --- | --- | --- | --- | --- |
| **Moderators** | ***k*** | ***N*** | ***r*** | ***ρ*** | ***SD*** | **L** | **U** | **L** | **U** | ***Q_M_*** | ***Q_E_*** | ***I^2^*** | ***R^2^*** |
| **Technology Context** | |  |  |  |  |  |  |  |  |  |  |  |  |
| Social media | 6 | 1,107 | .40 | .45 | .32 | .39 | .51 | -.08 | .98 | 5.18* | 4737.63** | 92.01% | .70% |
| Non-social media | 19 | 8,170 | .34 | .39 | .30 | .33 | .45 | -.11 | .89 |  |  |  |  |
| **Individualism vs. Collectivism** | | | |  |  |  |  |  |  |  |  |  |  |
| Individualism | 13 | 4,684 | .37 | .43 | .32 | .38 | .48 | -.09 | .95 | 2.71 | 2388.90** | 98.51% | 22.46% |
| Collectivism | 7 | 1,977 | .49 | .58 | .16 | .49 | .66 | .31 | .85 |  |  |  |  |
| **Power Distance** |  |  |  |  |  |  |  |  |  |  |  |  |  |
| High | 7 | 1,977 | .49 | .58 | .16 | .49 | .66 | .31 | .85 | 2.71 | 2388.90** | 98.51% | 22.46% |
| Low | 13 | 4,684 | .37 | .43 | .32 | .38 | .48 | -.09 | .95 |  |  |  |  |
| **Uncertainty Avoidance** | | |  |  |  |  |  |  |  |  |  |  |  |
| High | 9 | 4,344 | .36 | .41 | .22 | .25 | .58 | .06 | .76 | 109.01** | 1187.99** | 97.02% | 61.77% |
| Low | 11 | 2,317 | .50 | .59 | .29 | .56 | .63 | .11 | 1.00 |  |  |  |  |
| **Orientation** |  |  |  |  |  |  |  |  |  |  |  |  |  |
| Long-term | 15 | 5,761 | .36 | .43 | .21 | .30 | .55 | .09 | .77 | 457.96** | 583.06** | 93.99% | 81.57% |
| Short-term | 5 | 900 | .70 | .80 | .18 | .76 | .83 | .50 | 1.00 |  |  |  |  |
| **Indulgence vs. Restraint** | |  |  |  |  |  |  |  |  |  |  |  |  |
| Indulgence | 8 | 1,704 | .52 | .60 | .34 | .56 | .64 | .04 | 1.00 | 165.28** | 968.79** | 96.35% | 68.94% |
| Restraint | 12 | 4,957 | .37 | .43 | .20 | .30 | .57 | .11 | .75 |  |  |  |  |
| **Focal Variable** | | | | | | | | | | | | | |
| Quality | 7 | 3,355 | .34 | .40 | .24 | .16 | .63 | .01 | .79 | 30.50** | 3330.35** | 87.47% | 30.36% |
| Credibility | 8 | 1,577 | .28 | .31 | .36 | .25 | .37 | -.27 | .89 |  |  |  |  |
| Trust | 10 | 4,345 | .38 | .45 | .25 | .39 | .51 | .04 | .86 |  |  |  |  |
| **Sample Clinical Status** | | |  |  |  |  |  |  |  |  |  |  |  |
| Patients | 3 | 1,646 | .21 | .24 | .35 | -.23 | .71 | -.33 | .81 | .57 | 3933.17** | 98.86% | 17.65% |
| Non-patients | 22 | 7,631 | .38 | .44 | .30 | .39 | .49 | -.06 | .94 |  |  |  |  |
| **Sample Type** |  |  |  |  |  |  |  |  |  |  |  |  |  |
| Students | 6 | 1,133 | .46 | .53 | .29 | .48 | .58 | .06 | 1.00 | 4.69* | 4737.30** | 92.01% | .71% |
| Non-students | 19 | 8,144 | .33 | .39 | .31 | .32 | .46 | -.12 | .90 |  |  |  |  |
| **Study Method** |  |  |  |  |  |  |  |  |  |  |  |  |  |
| Survey | 22 | 8,870 | .34 | .40 | .29 | .34 | .47 | -.08 | .88 | 3.39 | 4716.90** | 91.95% | 1.14% |
| Experiment | 3 | 407 | .41 | .46 | .44 | .38 | .54 | -.26 | 1.00 |  |  |  |  |
| **Stimulus Type** |  |  |  |  |  |  |  |  |  |  |  |  |  |
| General | 11 | 5,688 | .28 | .32 | .28 | .15 | .49 | -.14 | .78 | 100.36** | 1815.40** | 97.52% | 62.28% |
| Specific | 14 | 3,589 | .46 | .54 | .30 | .51 | .58 | .04 | 1.00 |  |  |  |  |
| **Publication Year** |  |  |  |  |  |  |  |  |  |  |  |  |  |
| Prior to 2014 | 10 | 4,430 | .21 | .25 | .26 | .04 | .45 | -.17 | .67 | 146.34** | 2144.56** | 97.90% | 55.34% |
| 2014 and after | 15 | 4,847 | .47 | .55 | .25 | .51 | .59 | .14 | .96 |  |  |  |  |

*Note*. *k*=number of samples; *N*=total sample size; *r*=weighted mean correlation; *ρ*=weighted mean correlation corrected for measurement unreliability; SD=standard deviation of *ρ*; 95% CI=lower and upper limits of 95% confidence interval; 90% CV=lower and upper limits of 90% credibility interval; *Q_M_*=moderator test; *Q_E_*=amount of observed heterogeneity unexplained by the moderator; *I^2^*=percentage of variation across studies that is due to heterogeneity; *R^2^*=percent of variation explained by random-effects regression model.

***p*<.01, **p*<.05.
