# Supplementary material for: Consumers’ Evaluation of Web-Based Health Information Quality: Meta-analysis
Source: J Med Internet Res. 2022 Apr 28;24(4):e36463. doi: 10.2196/36463 (PMC9100526; doi:10.2196/36463)
Supplement: Multimedia Appendix 10 [file jmir_v24i4e36463_app10.docx]

**Multimedia Appendix 10. Influence of moderators on the relationship between web-based health IQ and behavior**

|  |  |  |  |  |  | **95% CI** | | **90% CV** | |  |  |  |  |
| --- | --- | --- | --- | --- | --- | --- | --- | --- | --- | --- | --- | --- | --- |
| **Moderators** | ***k*** | ***N*** | ***r*** | ***ρ*** | ***SD*** | **L** | **U** | **L** | **U** | ***Q_M_*** | ***Q_E_*** | ***I^2^*** | ***R^2^*** |
| **Individualism vs. Collectivism** | | | |  |  |  |  |  |  |  |  |  |  |
| Individualism | 20 | 33,688 | .23 | .27 | .29 | .09 | .46 | -.20 | .74 | 1.67 | 17399.54** | 99.52% | .57% |
| Collectivism | 9 | 5,515 | .31 | .37 | .27 | .14 | .59 | -.08 | .82 |  |  |  |  |
| **Power Distance** |  |  |  |  |  |  |  |  |  |  |  |  |  |
| High | 9 | 5,515 | .31 | .37 | .27 | .14 | .59 | -.08 | .82 | 1.67 | 17399.54** | 99.52% | .57% |
| Low | 20 | 33,688 | .23 | .27 | .29 | .09 | .46 | -.20 | .74 |  |  |  |  |
| **Uncertainty Avoidance** | | |  |  |  |  |  |  |  |  |  |  |  |
| High | 12 | 14,139 | .35 | .42 | .30 | .27 | .56 | -.07 | .91 | 2.02 | 11267.13** | 99.11% | 35.67% |
| Low | 17 | 25,064 | .17 | .21 | .28 | -.08 | .50 | -.25 | .67 |  |  |  |  |
| **Orientation** |  |  |  |  |  |  |  |  |  |  |  |  |  |
| Long-term | 17 | 13,738 | .24 | .29 | .25 | .05 | .53 | -.12 | .70 | .08 | 15923.18** | 99.44% | 9.02% |
| Short-term | 12 | 25,465 | .24 | .28 | .34 | .08 | .49 | -.28 | .84 |  |  |  |  |
| **Indulgence vs. Restraint** | |  |  |  |  |  |  |  |  |  |  |  |  |
| Indulgence | 14 | 22,776 | .16 | .20 | .28 | .01 | .38 | -.25 | .65 | .92 | 4403.16** | 99.27% | 33.88% |
| Restraint | 12 | 11,502 | .24 | .29 | .27 | .01 | .56 | -.15 | .73 |  |  |  |  |
| **Focal Variable** | | | | | | | | | | | | | |
| Quality | 6 | 4,095 | .34 | .40 | .30 | .16 | .63 | -.09 | .89 | 4.12 | 14316.30** | 99.34% | 18.24% |
| Credibility | 7 | 7,341 | .15 | .19 | .21 | .01 | .36 | -.16 | .54 |  |  |  |  |
| Trust | 17 | 27,889 | .25 | .30 | .30 | .11 | .48 | -.19 | .79 |  |  |  |  |
| **Sample Clinical Status** | | |  |  |  |  |  |  |  |  |  |  |  |
| Patients | 6 | 4,192 | .09 | .11 | .35 | -.32 | .53 | -.47 | .69 | .04 | 15754.41** | 99.55% | 10.30% |
| Regular | 25 | 35,319 | .26 | .31 | .27 | .14 | .47 | -.14 | .76 |  |  |  |  |
| **Sample Type** |  |  |  |  |  |  |  |  |  |  |  |  |  |
| Students | 9 | 7,008 | .56 | .66 | .37 | .57 | .76 | .05 | 1.00 | 3.71* | 3217.03** | 97.10% | 81.82% |
| Non-students | 22 | 32,503 | .17 | .20 | .25 | .06 | .35 | -.21 | .61 |  |  |  |  |
| **Stimulus Type** |  |  |  |  |  |  |  |  |  |  |  |  |  |
| General | 25 | 35,528 | .24 | .29 | .29 | .12 | .46 | -.19 | .77 | 1.04 | 16244.32** | 99.56% | 7.51% |
| Specific | 6 | 3,983 | .22 | .27 | .26 | .08 | .47 | -.15 | .69 |  |  |  |  |
| **Publication Outlet** |  |  |  |  |  |  |  |  |  |  |  |  |  |
| Journal | 26 | 38,726 | .24 | .29 | .29 | .12 | .45 | -.18 | .76 | .01 | 17285.59** | 99.61% | 1.57% |
| Non-journal | 5 | 785 | .19 | .22 | .29 | -.08 | .52 | -.25 | .69 |  |  |  |  |
| **Publication Year** |  |  |  |  |  |  |  |  |  |  |  |  |  |
| Prior to 2014 | 15 | 24,416 | .23 | .27 | .32 | .06 | .48 | -.25 | .79 | .00 | 15747.84** | 99.39% | 10.34% |
| 2014 and after | 16 | 15,095 | .26 | .31 | .26 | .09 | .53 | -.11 | .73 |  |  |  |  |

*Note*. *k*=number of samples; *N*=total sample size; *r*=weighted mean correlation; *ρ*=weighted mean correlation corrected for measurement unreliability; SD=standard deviation of *ρ*; 95% CI=lower and upper limits of 95% confidence interval; 90% CV=lower and upper limits of 90% credibility interval; *Q_M_*=moderator test; *Q_E_*=amount of observed heterogeneity unexplained by the moderator; *I^2^*=percentage of variation across studies that is due to heterogeneity; *R^2^*=percent of variation explained by random-effects regression model.

***p*<.01, **p*<.05.
